# Comparative genomics of wild type yeast strains unveils important genome diversity

**DOI:** 10.1186/1471-2164-9-524

**Published:** 2008-11-04

**Authors:** Laura Carreto, Maria F Eiriz, Ana C Gomes, Patrícia M Pereira, Dorit Schuller, Manuel AS Santos

**Affiliations:** 1 Departamento de Biologia & CESAM, Universidade de Aveiro, 3810-193 Aveiro, Portugal; 2 BIOCANT, Centro de Inovação em Biotecnologia, Parque Tecnológico de Cantanhede, Núcleo 04, Lote 3, 3060-197 Cantanhede, Portugal; 3 Centro de Biologia Molecular e Ambiental (CBMA) Universidade do Minho, Braga, Portugal

## Abstract

**Background:**

Genome variability generates phenotypic heterogeneity and is of relevance for adaptation to environmental change, but the extent of such variability in natural populations is still poorly understood. For example, selected *Saccharomyces cerevisiae *strains are variable at the ploidy level, have gene amplifications, changes in chromosome copy number, and gross chromosomal rearrangements. This suggests that genome plasticity provides important genetic diversity upon which natural selection mechanisms can operate.

**Results:**

In this study, we have used wild-type *S. cerevisiae *(yeast) strains to investigate genome variation in natural and artificial environments. We have used comparative genome hybridization on array (aCGH) to characterize the genome variability of 16 yeast strains, of laboratory and commercial origin, isolated from vineyards and wine cellars, and from opportunistic human infections. Interestingly, sub-telomeric instability was associated with the clinical phenotype, while Ty element insertion regions determined genomic differences of natural wine fermentation strains. Copy number depletion of *ASP3 *and *YRF1 *genes was found in all wild-type strains. Other gene families involved in transmembrane transport, sugar and alcohol metabolism or drug resistance had copy number changes, which also distinguished wine from clinical isolates.

**Conclusion:**

We have isolated and genotyped more than 1000 yeast strains from natural environments and carried out an aCGH analysis of 16 strains representative of distinct genotype clusters. Important genomic variability was identified between these strains, in particular in sub-telomeric regions and in Ty-element insertion sites, suggesting that this type of genome variability is the main source of genetic diversity in natural populations of yeast. The data highlights the usefulness of yeast as a model system to unravel intraspecific natural genome diversity and to elucidate how natural selection shapes the yeast genome.

## Background

The genome of wild-type and laboratory strains of *Saccharomyces cerevisiae *(yeast) has significant genetic variability. In general, natural isolates are often polyploid or aneuploid and have high degree of genetic variability and an essentially asexual life cycle [1-4]. Indeed, environmental perturbation often selects strains that display local gene amplifications, changes in chromosome copy number or gross chromosomal rearrangements, such as intra- or inter-chromosomal translocations, mediated by transposon-related sequences [5-7].

Recent comparative genomics studies showed that wild-type yeast strains cluster according to technological application rather than geographical distribution [8-10]. However, the species as a whole is not domesticated and consists of both wild-type and comercial populations. For example, specialized sake and wine strains were derived from natural populations not associated with alcoholic beverages, rather than the opposite [11]. Also, yeast strains are found in diverse habitats, namely in oak exudates [12,13], gut of insects [14], plant leaves and in grape berries [15]. Interestingly, damaged grape barriers, but not undamaged berries, are an important source of yeast strains [16]. The diversity of yeast strains in viticultural regions is rather high, suggesting the occurrence of specific natural strains associated with particular *terroirs *[17-21].

Simple sequence repeat (SSR) analysis, used to determine phylogenetic relationships between 651 yeast strains isolated from 56 worldwide geographical origins [22], showed that macro geographical differentiation of strains from Asia, Europe and Africa accounted for only 28% of the observed genetic variation, suggesting clonal reproduction and local domestication. The close association between vine migration and wine yeast favors the hypothesis that yeast may have followed man and vine as a commensal member of grapevine micro flora. SSRs were also used to distinguish populations from vineyards in close geographical locations and showed that genetic differences among yeast populations were apparent from gradations in allele frequencies rather than from distinctive "diagnostic" genotypes [23].

The continuous utilization of yeast strains for industrial purposes introduced artificial selective pressure that may have also influenced genome features and novel specialization routes. In fact, yeast has been identified as an emerging human pathogen that can cause clinically relevant infections in immune compromised patients [24,25]. Such pathogenic strains are phylogenetically related to baking strains, grow at higher temperature, produce extracellular proteases, are capable of pseudohyphal growth and may be resistant to antifungal treatment [10,26,27]. Also, the genome of the pathogenic *S. cerevisiae *strain YJM789 has very high percentage of sequence polymorphisms (60 000 SNPs scattered over the genome), which may be a primary cause of phenotypic variation [28].

The wide ecological, geographical, clinical and industrial distribution of yeast strains and the genome diversity already uncovered suggests that it is a good model system to understand genome diversity in natural populations and elucidate the relevance of such diversity for adaptation to changing environments and to new ecological niches. One of the first comprehensive studies on genetic variation of yeast strains, carried out using high-density oligonucleotide arrays containing up to 200,000 oligonucleotide probes from the yeast genomic sequence, unveiled differences at the level of single nucleotide polymorphisms and gene copy number alterations [29]. A similar approach revealed unexpected differences in 288 genes between the S288C and CEN.PK113-7D laboratory strains, involving differential gene amplification, gene absence or sequence polymorphisms [30].

In order to shed new light on the genome diversity of natural populations of yeast, we have isolated more than 1000 strains, genotyped them and identified clusters that distinguished the various genotypes. We then selected representatives of these clusters and compared their genomes with the genomes of clinical and commercial strains. For this, we used spotted DNA microarrays containing probes for the complete gene set of the S288C reference strain. We compared the genomes of five commercial winemaking strains, eight strains isolated from winemaking environments of two wine producing regions in Portugal, namely the Bairrada and Vinho Verde appellations of origin, and three clinical strains. The laboratorial strain S288C was used as reference for relative genome profiling. Our results highlighted differences between the laboratorial strain and the wild-type isolates linked to sub-telomeric instability and retrotransposon activity. These elements shaped differently the genomes of yeast strains from wine and clinical environments. The study also identified functional classes of genes where the copy number variations were associated with strains from wine- or clinical-related environments. No correlation was found between geographical origin and relative genome profile in the larger group of wine-related strains.

## Results

### Strains and overview of genomic variability

**Figure 1:**
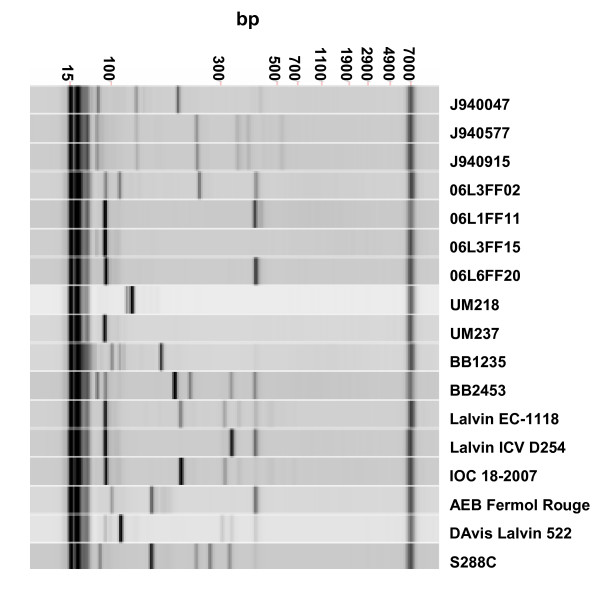
**Inter-delta region profiles**. The environmental, clinical and commercial yeasts used in this study were genotyped by PCR amplification of the inter-delta regions. The strains used in this study were selected from our yeast culture collection on the basis of their different inter-delta PCR profiles, as shown. Inter-delta PCR profiles were obtained by DNA electrophoresis on a Labchip HT (Caliper LS), and the data was displayed using the DataViewer software (Caliper LS). Each lane is identified at the top.

A total of 16 wild-type strains plus the reference S288C strain were used in this study (Table 1). The wine strains were selected amongst isolates of wine cellars and vineyards of Bairrada and Vinho Verde wine regions, in Portugal. Strains UM218 and UM237 were selected among 300 strains isolated from the Vinho Verde Region [19] for their distinctive genetic (simple sequence repeats) and enzymatic (API ZYM system) profiles. Strains from the Bairrada region were selected from 800 isolates collected during two consecutive years of grape harvest and wine production and represented major strain clusters of inter-delta region PCR genotyping profiles (A. C. Gomes, unpublished) (Figure 1). Commercial yeast strains used in industrial wine must fermentations were Lalvin EC-1118, used in the Vinho Verde region, and Lalvin ICV D254, IOC 18–2007, AEB Fermol Rouge and Davis Lalvin 522, commonly used in the Bairrada region. These commercial strains were initially selected from French wine producing regions (Table 1) and are used world-wide. Finally, three strains, isolated from patients suffering from opportunistic fungal infections were also included in this study. These strains were selected from a clinical strain collection on the basis of their inter-delta genotyping profiles (Figure 1). The inter-delta profiles of J940915 and J940557 were very similar and these strains were used to ascertain how the inter-delta region relatedness, used to genotype the strain collection, correlated with the genomic variability.

**Table 1:** Yeast strains used in this study

**Strains**	**Characteristics**	**Source/origin**
J940047	Wild-type isolate	Clinical, Portugal
J940557	Wild-type isolate	Clinical, Portugal
J940915	Wild-type isolate	Clinical, Portugal
06L3FF02	Wild-type isolate	Wine cellar; Bairrada Wine Region, Portugal
06L1FF11	Wild-type isolate	Wine cellar; Bairrada Wine Region, Portugal
06L3FF15	Wild-type isolate	Wine cellar; Bairrada Wine Region, Portugal
06L6FF20	Wild-type isolate	Wine cellar; Bairrada Wine Region, Portugal
UM218	Wild-type isolate	Vineyard; Vinho Verde Wine Region, Portugal
UM237	Wild-type isolate	Vineyard; Vinho Verde Wine Region, Portugal
BB1235	Wild-type isolate	Vineyard; Bairrada Wine Region, Portugal
BB2453	Wild-type isolate	Vineyard; Bairrada Wine Region, Portugal
Lalvin EC-1118	Commercial; used in the Vinho Verde Wine Region	Champagne, France
Lalvin ICV D254	Commercial; used in the Bairrada Wine Region	Rhône Valley, France
IOC 18–2007	Commercial; used in the Bairrada Wine Region	Institut Oenologique de Champagne, France
AEB Fermol Rouge	Commercial; used in the Bairrada Wine Region	Montpellier University, France
Davis Lalvin 522	Commercial; used in the Bairrada Wine Region	University of California, Davis, USA
S288C	MATα SUC2 *mal mel gal2 CUP1 flo1 flo8-1*	Mortimer & Johnston, 1986 [87]

Since natural hybridization between species of the *Saccharomyces sensu stricto *group can occur [31] and, indeed, several *S. cerevisiae *× *S. kudriavzevii, S. bayanus *× *S. cerevisiae *and *S. bayanus *× *S. cerevisiae *× *S. kudriavzevii*, have been described among wine strains [32-36], all strains were tested for their hybrid nature. For this, *MET2 *locus restriction fragment analysis was used. *S. cerevisiae*-specific profiles identical to those of the reference strain S288C were detected in all cases, suggesting that the strains selected for aCGH analysis were authentic *S. cerevisiae*. These results were corroborated by an additional analysis of 10 polymorphic *S. cerevisiae*-specific simple sequence repeats (results not shown). Finally, PCR-RFLP profiling of the *OPY1, KIN82, MET6, KEL2 *and *CYR1 *loci, which are located on chromosomes II, III, V, VII and X, respectively, confirmed the above data, thus unequivocally demonstrating that the strains used had a *S. cerevisiae*-specific profile and were not hybrids (Additional File 1, Figure S1).

For aCGH analysis, genomic DNA from each strain was fluorescently labeled and competitively hybridized with genomic DNA from the reference strain S288C, with duplicate experiments in reverse Cy-dye labeling (dye-swap) design (see Methods).

**Figure 2:**
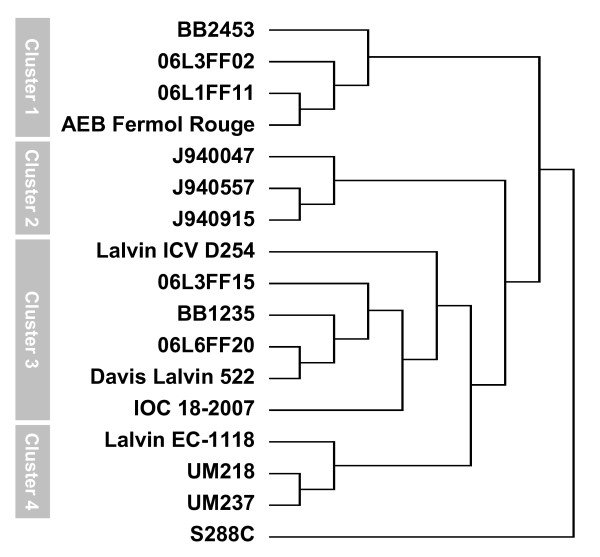
**Hierarchical clustering of aCGH profiles**. The strains used in his study were grouped according to their aCGH profiles. For this, hierarchical clustering analysis, using Pearson correlation with average linkage, of the normalized aCGH profiling data was performed. The clusters shown identify strains that shared similar ORF copy number alterations.

Hierarchical cluster analysis of the aCGH data showed high genome variability (Figure 2), and the laboratory strain S288C was clearly differentiated from the other 16 strains. Clinical strains (Cluster 2) were clearly differentiated from the wine strains clusters (1, 3 and 4). High similarity between the genomes of two of the clinical strains, J940557 and J940915 was expected from their identical inter-delta region profiles (Figure 1) and microsatellite patterns (data not shown), and such genome similarity was confirmed by aCGH profile similarity (Figure 2). Other pairs of strains with distinct inter-delta region profiles showed the high similarity in the clustering tree, namely, AEB Fermol Rouge and 06L1FF11, Davis Lalvin 522 and 06L6FF20 or UM218 and UM237.

Environmental and commercial wine strains formed three clusters (Cluster 1, 3 and 4) and differences between them were as high as those observed between wine and clinical strains. Divergence among wine yeast was not correlated to geographical origin, since strains BB1235 and 06L6FF20 (Cluster 3, Bairrada region) were more similar to strains UM218 and UM237 (Cluster 4, Vinho Verde region) than to strains BB2453, 06L3FF02 and 06L1FF11 (Cluster 1, Bairrada region). Furthermore, commercial strains isolated from French winemaking regions grouped together with strains from the Bairrada and Vinho Verde regions. There was also no separation of strains isolated from vineyards (BB2453, BB1235, UM218 and UM237), from cellars (06LF3FF02, 06L1FF11, 06L6FF20, 06L3FF15) or from commercial strains (AEB Fermol Rouge, Lalvin ICV D254, Davis Lalvin 522, IOC18–2007, Lalvin EC-1118).

### Genome variability is associated with Ty elements and telomeres

Yeast genomes evolve through dynamic processes and often contain gene duplications and deletions or even chromosomal segment rearrangements. Genome instability occurs throughout the genome, but is more frequent in particular regions, such as near Ty elements, due to reciprocal translocations, or at sub-telomeric regions, possibly caused by high-frequency of ectopic recombination [37]. To investigate the presence of regions of increased genome variability we used the "Cluster Along Chromosomes" (CLAC) method of the CGH Miner software package [38]. This software highlighted the occurrence of clusters of altered data in a set of samples relatively to controls, using a moving data window to calculate an average log ratio value, while controlling the False Discovery Rate (FDR), according to user defined parameters. Karyoscope maps that indicated the location of regions with alterations in ORF copy number (amplifications and deletions) for each strain were obtained using CGH Miner analysis as previously described by Dunn and colleagues [39], with the chromosomal coordinates of S288C genome. Since some aCGH probes interrogated duplicated or multicopy ORFs, namely ORFs of Ty elements, those karyoscope maps display the average dosage of the repeated ORFs rather than the dosage of each of the repeated ORFs. A moving window of three ORFs was chosen as the lowest statistically meaningful interval for averaging the hybridization signal, thus defining the resolution of the analysis.

**Figure 3:**
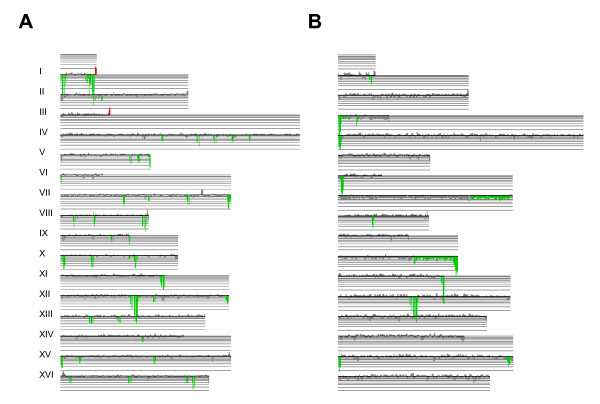
**Karyoscope maps of strains Lalvin ICV D254 (A) and J940915 (B)**. In order to visualize the gene copy number alterations along chromosomes and to have a global overview of the alterations detected by aCGH, the data was plotted along each chromosome, using the annotated ORF coordinates of S288C. Vertical bars represent the relative hybridization pattern relatively to the genome of strain S288C. Red bars correspond to amplified ORFs, green bars represent deleted ORFs and grey bars are statistically non-significant alterations (FDR <0.279). The horizontal lines indicate the hybridization ratios in logarithmic scale. Signals of repeated ORFs, such as those of Ty elements or repetitive sequences flanking Ty element insertion sites, correspond to the average signal rather than the individual signal dosage of that sequence. Clusters of altered ORFs contained roughly one third of Ty -ORFs and contributed to localization of the chromosome alterations following the genome coordinates of the S288C strain.

**Figure 4:**
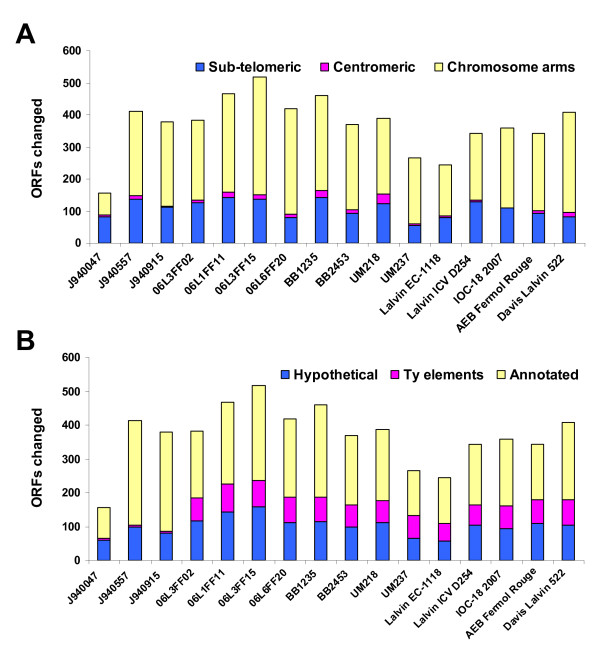
**Chromosomal location and classification of ORFs with variable copy number**. Panel-A shows the distribution of variable ORFs indicated by CGH-Miner in terms of chromosome location, that is, sub-telomeric, centromeric or chromosome arms. Panel-B shows the distribution of the same ORFs in terms of abundance of Ty elements, hypothetical or annotated ORFs. In both panels, each bar represents the total number of variable ORFs for a given strain.

The karyoscope maps displaying the relative hybridization data derived for each strain revealed that the majority of the genome alterations corresponded to deletions relative to strain S288C, while ORF amplifications were rare (Figure 3 and Additional File 2, Figures S2A–S2P). ORF amplification clusters were found in some strains, mostly located in sub-telomeric regions (Figure 3A) and within 20 Kb of the S288C respective chromosome end, but only few corresponded to genes with annotated function. Clusters of depleted ORFs were found in sub-telomeric regions and contained large percentage of Ty elements and hypothetical ORFs (Figure 4). In the group of wine strains, up to one third of the observed gene copy number alterations were found in sub-telomeric regions (Figure 4A) – within 50 Kb from the S288C chromosome ends, using the criterion of Edwards-Ingram and colleagues [40]. On average, the clinical strains showed slightly higher percentage (almost 40%) of depleted ORFs localized near the telomeres. However, this is mostly explained by the massive loss of chromosomes VII and X right arms in strains J940557 and J940915 (see karyoscope map of J940915 in Figure 3B). The depletion of ORFs around the centromeric regions- within 20 Kb of the centromere, according to the criterion of Schacherer and colleagues [41]- was reduced and only slightly above average in some of the wine strains.

A clear differentiation between clinical and wine related strains was observed when the frequency of transposable elements within the ORFs with depleted copy number (Figure 4B) was considered. Ty elements comprised 36% of the ORFs absent in all wine strains, which meant that approximately one third of the ORFs associated with retrotransposon activity (42 out of a total of 113) in S288C were absent in these strains. The genomes of the clinical strains, however, contained most of the Ty elements identified in the genomic sequence of S288C, since only 7% of the ORFs absent in all three of them were classified in this category.

### Highly variable genomic regions in wine and clinical strains

**Figure 5:**
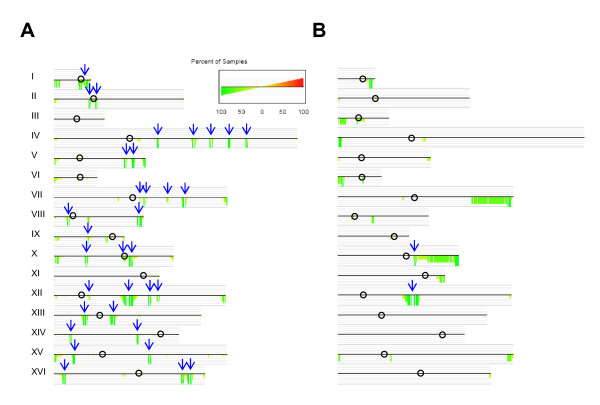
**The ORF variability patterns differ between wine and clinical strains**. The consensus karyoscope maps obtained for wine (Panel-A) and clinical (Panel-B) strains showed that ORF variation in wine strains was distributed along chromosomes while in clinical strains such distribution was more frequent in sub-telomeric regions. The consensus maps show the relative percentage of strains (percent of samples) within wine (Panel-A) and clinical (Panel-B) environments with copy number alteration in a given ORF, relatively to the reference strain S288C. The data was plotted according to ORF chromosome location. Red bars represent percentage of strains with amplifications and green bars represent the percentage of strains with deletions, according to the grey-line scale shown above and below the chromosome central line. Open circles indicate the position of centromeres and blue arrows identify Ty elements.

The differences between wine and clinical strains were further highlighted with the consensus plots obtained from the intersection of the individual karyoscope maps of the thirteen wine fermentation strains (Figure 5A), compared to the three clinical isolates (Figure 5B). These plots represented the relative amount of samples with copy number alterations, showing the percentage of strains with a given amplification or deletion. Most of the deletion clusters identified in wine strains co-localized with absent Ty elements (Figure 5A), while in clinical isolates a significant number of depletions were associated with sub-telomeric instability (Figure 5B). Variable regions affected different sub-functional categories of genes in wine and clinical strains. For example, carbohydrate transporters and glycosidases were the main functional groups of genes absent in clinical strains, but genes involved in vesicle transport, telomere maintenance and alcohol metabolism were also identified among the lost sub-telomeric genes. The functional categories most affected by gene copy number variability in wine strains were related to vitamin metabolism, DNA recombination, polysaccharide metabolism, regulation of meiotic cell cycle and reproduction, and were frequently located in the vicinity of Ty element insertion sites.

### *ASP3 *and *YRF1 *gene families differentiate wild-type and laboratory strains

**Figure 6:**
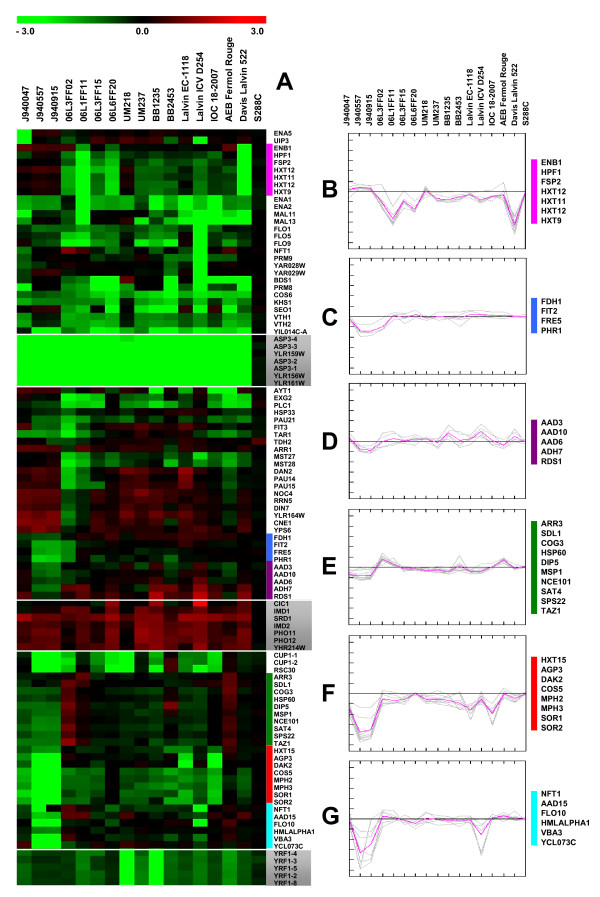
**Gene copy number alterations show significant inter-strain variability**. Several genes were deleted in all environmental, clinical and commercial strains, while other genes were amplified in almost all strains, relative to the laboratory S288C strain (boxed blocks). However, most of the genes showed significant copy number variability between strains. Panel-A represents the aCGH values (M values) for genes with altered copy number in at least one of the strains. The genes with similar copy number alterations in the wild-type strains relatively to the reference laboratorial strain S288C were highlighted in grey (boxed blocks). The coloured bars next to the gene names identify groups of genes whose relative hybridization value is shown in the line graphs of Panels B-G. In these panels, grey lines represent the aCGH values of genes indicated on the right hand side of each panel (also shown in the color coded map of Panel-A) and the pink line represents the average aCGH values of the genes (lines).

The karyoscope maps constructed by the moving average window algorithm (implemented in CGH miner) highlighted genes with copy number alterations. Of the 634 ORFs identified as altered by the CLAC algorithm, in at least one of the strains, significance analysis confirmed 270 ORF alterations. From these, 30% corresponded to Ty elements, mostly located in depleted hybridization clusters in wine strains (Figure 5). Another third of ORFs with copy number variation were hypothetical ORFs and were contiguous to deleted Ty elements in wine strains. Annotated variable ORFs constituted the remaining third of significantly altered ORFs and the respective aCGH values (M values) were depicted in Figure 6A for further discussion.

General trends in copy number alterations included both amplified and depleted genes. Genes with the same trend in copy number alteration across all strains were highlighted in grey. Among the genes depleted in all strains, relative to the reference strain S288C, were four copies of tandemly-repeated cell-wall asparaginase genes (*ASP3-1, ASP3-2, ASP3-3 *and *ASP3-4*), which are induced in response to nitrogen starvation [42]. Also, ORFs YLR161W, YLR156W and YLR159W, that code for putative proteins of unknown function and of identical sequence, were depleted in all strains. These genes are located in the right arm of Chromosome XII near a ribosomal DNA region, in a chromosome locus corresponding to a large cluster of depleted ORFs flanking Ty elements, and were indicated in the karyoscope consensus maps of both wine and clinical strains (Figure 5A, B).

The relative hybridization values of significantly altered genes (Figure 6A) identified another set of homologous genes whose copy number was altered in all wild-type strains. Indeed, five genes of the *YRF1 *family, which are located in telomeric Y' elements and encode DNA helicases (*YRF1-2*, *YRF1-3*, *YRF1-4*, *YRF1-5 *and *YRF1-8*), were present in the genome of S288C, but were depleted or, at least, partially depleted in environmental, clinical and commercial strains. The missing *YRF1 *genes were part of the depleted ORF clusters located at the telomeres of the right arms of chromosomes V, VII, XII and XV (Figure 5), together with several hypothetical ORFs.

Gene YIL014C-A, coding for a putative protein of unknown function, was deleted in all strains except in J940047. The same was observed for the tandem repeated genes *ENA1 *and *ENA2*, which code for a P-type ATPase sodium pump involved in the efflux of sodium and lithium ions, required for salt tolerance, which were depleted in all strains except 06L3FF02. Gene *ENA5 *was only depleted in strain J940047.

A group of genes with increased copy number in almost all strains was also identified (Figure 6A). Among them, *IMD1*, *IMD2*, *PHO11*, *PHO12 *were signaled by the CLAC algorithm as belonging to clusters of sub-telomeric genes amplified in strains Lalvin EC-1118, Lalvin ICV 254 and UM237, but aCGH values suggested that they could also be amplified in other strains (Figure 6A). *PHO11 *and *PHO12 *genes, which code for acid phosphatases and are induced by phosphate starvation, increased their copy number by a factor of 3 in strain UM237 relative to S288C, while in strain Lalvin EC-1118 the fold increase in hybridization signal was more compatible with its duplication. The copy number of the *IMD2 *gene increased by a factor of 2 in Lalvin EC-1118 and UM237 strains, as well as in strain Lalvin ICV D254, although the latter was not highlighted as duplicated by CGH-Miner. In strain Lalvin ICV D254, *RDS1 *(a zinc cluster transcription factor involved in resistance to cycloheximide) was part of an amplified cluster of genes on Chromosome III and the aCGH signal indicated a 3-fold increase relative to S288C. The aCGH values for this gene (Figure 6A) further suggested that it was also amplified in other strains (see Panel D of Figure 6).

Interestingly, both *IMD1 *and *IMD2 *genes code for inosine monophosphate dehydrogenase and confer resistance to mycophenolic acid, which is produced by the fungus *Penicillium stoloniferum *and inhibits *de novo *purine synthesis. These genes, together with *RDS1*, are involved in resistance to compounds that inhibit eukaryotic cell proliferation and increased copy numbers point to a survival advantage in competitive ecosystems in presence of organisms that secrete mycophenolic acid or cycloheximide, as growth inhibitors.

### Gene copy number alterations and the origin of strains

Several genes showed different copy number alterations in wine and in clinical strains (Figure 6, panels B-F). For example, the genes *HXT9*, *HXT11 *and two ORFs of *HXT12 *(YIL170W and YIL171W) were depleted in most wine strains, with the exception of UM218, Lalvin EC-1118 and AEB Fermol Rouge, but did not show copy number variation in the clinical strains (Figure 6B). These genes code for putative hexose transporters which are non-functional in strain S288C. Similar copy number changes across the analyzed strains were identified for *HPF1 *and *FSP2*, which code for proteins with glucosidase activity: *HPF1 *codes for a haze-protective mannoprotein that reduces the particle size of aggregated proteins in white wines, while *FSP2 *is induced under nitrogen limitation. Also, the gene *ENB1*, which codes for a trans-membrane iron transporter of the major facilitator superfamily and is expressed under iron deprivation conditions, was included in this group. The absence of these genes was particularly notorious in strains isolated from the Bairrada region and in the commercial strain Davis Lalvin 522 (commonly used in Bairrada wine musts fermentations).

The genes *FDH1*, *FIT2*, *FRE5 *and *PHR1 *were absent in the clinical isolates J940557 and J940915 and in strain 06L3FF02. *FDH1 *codes for a formate dehydrogenase, *FIT2 *codes for a mannoprotein involved in the retention of siderophore-iron in the cell wall, *FRE5 *codes for a putative ferric reductase induced by low iron levels, and *PHR *codes for a DNA photolyase induced by DNA damage (Figure 6, panel C). These genes do not share a common function, but belong to a set of contiguous ORFs located in the sub-telomeric region of the right arm of Chromosome V, which was deleted in many of the strains (CLAC algorithm). This cluster of deleted ORFs included *FIT3*, which was also deleted in the above mentioned strains and in 06L1FF11, but showed aCGH values compatible with amplification of copy number in many of the natural wine strains and in J940047. Although redundant, considering the existence of other functional homologues, variability in copy number of these genes may affect the capacity of yeast to thrive in environments with high levels of exogenous formaldehyde, produced from plant material or from degradation of environmental pollutants, and in environments with low iron availability.

A group of genes coding for proteins with alcohol dehydrogenase activity were missing in strains J940557 and J940915, but were amplified in many of the wine strains (Figure 6, panel D). For example, *ADH7 *and *AAD3*, which belong to a cluster of amplified ORFs identified in Chromosome III in strain Lalvin ICV D254 by CLAC analysis (Figure 3A), showed increased copy number in strains BB1235 and Davis Lalvin 522. *AAD6 *and *AAD10 *were depleted in strains J940557 and J940915. Since these genes code for proteins involved in ethanol tolerance, their increase in copy number may confer high resistance to ethanol, which is one of the most important phenotypes for grape must fermentation. *RDS1 *was also included in this group of genes because of the similar copy number variation profile within the analyzed strains, probably due to its location in the same cluster of altered ORFs as *ADH7 *and *AAD3*, in Chromosome III.

A large set of genes were consistently deleted in the three clinical isolates (Figure 6A, panels E and G). The *COG3*, *HSP60*, *MSP1*, *NCE101 *genes (Figure 6, panel E) are implicated in protein transport and localization, while *ARR3 *and *DIP5 *are arsenite and amino acid transporters, respectively. These genes that were absent in clinical strains were amplified in some of the wine strains, namely 06L3FF02, 06L1FF11 and AEB Fermol Rouge. The relative abundance of these genes in the latter strains explained, at least in part, why their genome profiles differed from those of the other wine strains (Figure 2).

Other genes were absent in clinical and in many wine strains (Figure 6, panel F). For example, genes involved in carbohydrate transport (*HXT15*, *MPH2*, and *MPH3*) and sugar metabolism (*SOR1*, *SOR2*) belonged to this category. Strains Lalvin EC-1118 and IOC 18–2007 were the only wine strains with a decreased copy number in *AGP3*, which codes for an amino acid permease, and *DAK2*, participating in the glycerol catabolic process, while strain 06L6FF20 constituted an exception within the wine strains, since it showed identical copy number of all of these genes relatively to strain S288C.

### Gene copy number signatures for wine must fermentation

Many of the variable genes coded for transporters, permeases, or flocculation proteins, contributing to a genomic signature of the wine strains. Among these genes, the maltose transporter gene, *MAL11*, and the MAL-activator protein gene, *MAL13*, were particularly interesting because they are important for maltose assimilation and *MAL13 *is non-functional in the laboratory S288C strain [43]. These genes were absent in the commercial wine strains and in some of the environmental isolates. Also, only some of the commercial wine strains (Lalvin EC-1118 and AEB Fermol Rouge), showed aCGH values compatible with copy number increase of *CUP1-2*. Genes involved in flocculation mediated by cell wall protein-carbohydrate interactions, namely *FLO1*, *FLO5*, *FLO9 *and *FLO10*, were in general depleted in the wine strains, and also in two of the clinical strains. Evidences for variability in copy number of the homologous genes were found in almost all strains, with exception of Lalvin ICV D254, where the aCGH values indicated that these genes were deleted. Finally, copy number variability among wine strains was also found in genes *PAU14*, *PAU15 *and *PAU21 *that code for hypothetical proteins with structural similarity to the seripauperin family.

## Discussion

### aCGH profiles grouped yeast strains from different geographical origins

The genome variability uncovered within the environmental, clinical and commercial strains was pronounced and did not show any correlation between genome characteristics and ecosystem or geographical origin. Interestingly, this study unveiled high genomic similarity between the commercial strains and isolates from regions where these commercial strains were used in wine must fermentation. For instance, strains UM218 and UM237 from the Vinho Verde region share a similar genome hybridization profile (Figure 2) and grouped with strain Lalvin EC-1118 (Cluster 4), which is frequently used in the production of sparkling Vinho Verde. Strain IOC 18–2007, which is used in bottle-fermentations, is widely used in the Bairrada sparkling wine production, and grouped with some of the Bairrada isolates (Cluster 3). Close associations were also found between AEB Fermol Rouge and strains 06L1FF11, 06L3FF02, and BB2453, from cellars and vineyards of the Bairrada region, respectively (Cluster 1). Strains 06L6FF20, 06L3FF15 and BB1235 are more related to Davis Lalvin 522 than to any other commercial strain. AEB Fermol Rouge, isolated in France, and Davis Lalvin 522, obtained from the USA (Table 1), are both used in the Bairrada cellars to ferment wine musts due to their high fermentation performance with grapes from this region.

The continuous use of commercial yeast strains in large quantities and during successive years may lead to some genetic homogenization of resident strains that belong to the autochthonous yeast flora. This is supported by the fact that strain UM218 was collected in 2001, 300 meters from a winery where strain Lalvin EC-1118 was used in that year, whereas strain UM237 was collected, in 2003, 20 meters from a winery where the same commercial strain was used since 2001 [44]. However, since the Bairrada isolates come from cellars where no commercial strains were used, and the vineyards were not located in the vicinity of cellars (A. C. Gomes, personal communication), a contamination with commercial yeast does not explain the similarities between environmental isolates and some commercial strains. Instead, the winemaker's experience for the production of wines with the most desirable expression of the region's *terroir *may, indirectly, be responsible for this genomic resemblance. By favoring phenotypic characteristics in the autochthonous strains, which adapted during centuries of positive selection by farming and wine producing activities, the wine producer may have selected commercial yeast according to the same required phenotypes, and this resemblance in phenotype is reflected, to some extent, in similar gene copy number characteristics.

### Large scale genome alterations

*S. cerevisiae *strains that were mainly obtained from winemaking environment, are homothallic, mostly homozygous (65%), with low to high (> 85%) sporulation ability and are predominantly diploid [31,45-47]. Aneuploid strains have also been described [4,22,48,49]. Although no evidence for polyploidy was detected in the strains analyzed in this study, the karyoscope maps of the clinical isolates J940557 and J940915 (see Figure 3B) showed a systematic depletion in hybridization signal throughout the entire length of chromosomes III, VII and X. This depletion was only statistically significant at the right arm-ends, ruling out the hypothesis that these strains were aneuploid for the referred chromosomes. Also, no evidence for aneuploidy was found in the other strains.

One explanation for the karyoscopes of strains J940557 and J940915 may be the presence of heterologous chromosomes originated from a different strain, albeit similar to *S. cerevisiae *S288C. The latter hypothesis is supported by the frequent occurrence of *Saccharomyces *sp. hybrids, which originate from mating of different *Saccharomyces *species that form viable, although sterile, zygotes [31]. These hybrids are sometimes selected for industrial beer or wine fermentations due to phenotypic advantages [32,50,51], but inter-specific hybrids were also found in diverse spontaneous fermentations [33,52]. However, the strains used in our study, including the clinical isolates, were not hybrids (Additional File 1, Figure S1). Therefore, differences in chromosome copy number, that is, structural heteromorphisms, of chromosomes III, VII and X, may be the explanation for the obtained patterns of relative hybridization.

### Specific trends of genome instability distinguished wine and clinical isolates

Consensus maps highlighted the most variable regions of the yeast genome, relatively to the laboratory S288C strain. High variability was associated with the sub-telomeric regions of some chromosomes, irrespective of the strain's origin. In particular, a large fraction of strains, either from wine or clinical background, had ORF deletions located at the right end of Chromosome I, left end of Chromosome VI, and right end of chromosomes VII and X (Figure 5). Comparative genome studies performed by Winzeler and colleagues [29] showed that inter-species genome variability is biased toward sub-telomeric regions, where genes related to carbon source metabolism and transport are located. Apparently, telomere variability is important for adaptation to new environments and to different metabolic sources to overcome environmental stress.

Retrotransposons are also known as regions of high genome diversity between yeast strains and species [53,54]. Our data further supports the hypothesis that they may be selected for generating genomic variability in response to environmental stimuli, since they were affected differently in wine and clinical strains (Figure 5). Wine must fermentation isolates differed dramatically in Ty element composition from the reference laboratorial strain, as indicated by the relative absence of about one third of these elements, together with ORFs flanking their insertion sites. On the other hand, clinical strains were similar to S288C in composition of Ty element and Ty element associated ORFs.

This raised the question of whether clinical strains and S288C share a common ancestor or whether the reduced number of Ty elements and the flanking genes in the wine strains could result from selective pressures that affect particular regions of the genome in response to adaptation to particular environments. Genome comparison between the clinical isolate YJM789 and S288C undertaken by Wei and colleagues [28], showed a close association between repeat sequences sites in S288C and deleted regions in YJM789.

A comparison of the consensus plots of the strains used in this study with the sequences of the chromosomes of YJM789, showed that the clusters of depleted ORFs associated with Ty elements in the former (Figure 5A) were also missing in the YJM789 genome (see Additional File 3, Figures S3A–S3P). On the other hand, the consensus plot obtained for the clinical strains coincided with the comparative analysis carried out by Wei and colleagues [28]. In particular, in the sub-telomeric variability found in several chromosomes and in the clusters of deleted ORFs associated with Ty element insertion sites in chromosomes X and XII (Figure 5B). Since YJM789 is not a wine fermenting strain, but is associated to a pathogenic phenotype, the resemblance of genomic alterations related to Ty element content between this strain and those isolated from vineyards was surprising and deserves further study.

Variability in copy number of transposable elements has been previously reported within the Hemiascomycetous yeast clade [55], and particularly in this group of organisms [40]. This is in line with previous studies that showed low copy number of these elements relative to S288C in wild-type wine, lager and "flor" yeast [39,56,57], as well as in laboratory strains other than S288C [29]. The high variability in Ty element composition observed in this study supports the hypothesis that retrotransposition is relevant for adaptation, at least in the *Saccharomyces sensu stricto *clade. Sequences flanking transposable elements generate variability in the yeast genome [6], probably due to the ectopic recombination involving Ty element repetitive sequences, since Ty elements play a role in the mobilization of genome fragments throughout the genome, resulting frequently in chromosomal rearrangements and gene duplications [58]. Variability of Ty element content is supported by retrotransposon activity loss or acquisition in some lineages of *Saccharomyces sensu stricto*, correlating with speciation, according to Liti and colleagues [59]. Interestingly, these authors carried out a population survey of LTR-retrotransposons in the *Saccharomyces sensu stricto *complex and showed that Ty elements may be absent in groups of geographical isolates and are often lost or horizontally transferred in an apparent homeostatic control of the total number of repetitive elements.

### Gene copy number alterations differentiate laboratorial and environmental strains

The absence of the tandem repeated *ASP3 *region, located in Chromosome XII, as well as the *ENA *region of Chromosome IV, were observed both in wine and clinical strains (Figure 6). Nevertheless, such deletions were found in various strains and are not specific of wine or clinical phenotypes [29,40,56,60,61]. Variability in copy number of the *ASP3 *genes was found in a comparative hybridization study of 9 strains isolated from ale and lager brewing fermentations, suggesting that the presence or absence of these genes could discriminate fermentative yeast [62]. However, the absence of these genes in the wine yeasts analyzed in this study, including commercial and clinical isolates, only discriminated the laboratorial strain S288C from the environmental isolates, indicating that the use of asparagine as an alternative nitrogen source is not important in natural niches from were the wild-type strains were isolated.

Depletion of the genes of the *YRF1 *family also differentiated the environmental from the laboratorial reference strain. These DNA helicase genes are induced in strains with deficient telomerase activity, as part of a mechanism of telomere rescue [63]. These genes are present in sub-telomeric Y' elements, but seem to be dispensable, since a study on the survival and fitness of an *S. paradoxus *isolate without telomerase and in the absence of Y' elements was similar to that of other well characterized strains [59]. Although Y' elements are conserved between strains and species of *Saccharomyces *[64,65], copy number of the YRF1 family of genes varied remarkably in the group of strains surveyed in this study (Figure 6).

### Variation in fermentation related genes

Genes involved in maltose metabolism, namely *MAL11 *and *MAL13*, were depleted relative to S288C in most commercial wine strains, while some of the non-commercial isolates showed deletion of both or just one of these genes. In a similar genomic profile study of commercial wine strains, Dunn and colleagues [39] found intra-strain copy number variation of *MAL11 *and *MAL13 *genes and did not include them in their "commercial wine yeast genome signature". This signature also highlighted the deletion of two copies of *CUP1 *relatively to the genome of S288C. However, they found variability in copy number of these genes within isolates of the same commercial wine strain. Similarly, these genes were deleted in most of the wine related strains studied here, but not in BB2453 and AEB Fermol Rouge in which *CUP1-2 *was apparently amplified. These genes code for a protein that binds copper and mediates resistance to high concentrations of copper and cadmium and this locus is variably amplified in different yeast strains [66,67] and is not exclusive of wine fermentation strains.

Variability of copy number among the wine strains was also found in genes of the seripauperin family, namely *PAU14*, *PAU15 *and *PAU21*. These genes are encoded mainly in subtelomeric regions and are strongly regulated by anaerobiosis during alcoholic fermentation [68,69]. They play a role in sterol lipid transport and hence their relevance for ethanol tolerance during anaerobic growth. While *PAU21 *was depleted in all wine strains relative to S288C, *PAU14 *and *PAU15 *copy number increased in some of the wine strains, as well as in all clinical isolates, but decreased in others, namely in isolates whose genomic profiles were similar to those of AEB Fermol Rouge. *PAU15 *is involved in the response to toxins [70], but no function is yet known for *PAU14*.

## Conclusion

Our data showed that telomeric recombination and Ty element insertion were the main genome diversity features of the clinical, commercial and environmental isolates used in this study. Among the variable genes, mostly depleted, were genes involved in metabolic functions related to cellular homeostasis or transport of different solutes such as ions, sugars and metals. Clusters of depleted ORFs also contained ribosomal proteins, general transcription factors and transcription activators, translation initiation factors, helicases and zinc-finger genes.

In the clinical strains, genes associated to pathogenesis, namely those involved in pseudohyphal growth and invasiveness, did not show copy number alterations. This confirmed previous studies by Klingberg and colleagues [71], who did not find specific virulence factors separating clinical from non-clinical yeast strains. Despite this, Llanos and colleagues [27] found that clinical isolates have a typical pathogenic phenotype when compared with industrial yeasts. For example, secretion of proteases and phospholipases, growth at 42°C and pseudohyphal growth are more pronounced in clinical isolates. Some strains isolated from infections may be opportunistic colonizers of the human environment and not commensals of humans. Indeed, a recent survey of 92 yeast invasive infections revealed that 50% of them were caused by *Saccharomyces boulardii*, which is used as a probiotic preparation for the treatment of antibiotic-related diarrhea [72].

The genomic variability found in this study supported other studies showing duplication and deletion of sub-telomeric genes involved in secondary metabolism linked to environmental adaptation [73]. In this study, the sub-telomeric genes whose copy number changed, namely *MAL*, *FLO*, *HXT*, *PHO*, *IMD*, *SOR*, *PAU*, *FIT *and *ARR *family genes, did not identify specific alterations of environmental or of commercial wine strains. Also, our list of genes with variable copy number showed differences with the "commercial wine yeast signature" published by Dun and colleagues. [39]. However, some of the genes identified in our study confirmed the trend of genome alterations of wine strains highlighted by the "commercial wine yeast signature". For example, the *IMD *and *PHO *genes were amplified and the *MAL *genes were deleted in both studies. Genome variability associated with retrotransposon mobility was characteristic of wine strains. Therefore, these variability mechanisms may have a positive impact on the fitness of strains during colonization of new environment(s). In other words, this present study highlights the usefulness of yeast as a model system to study genomic variability in the context of environmental and evolutionary genomics.

## Methods

### Strains and culture conditions

A list of strains used in this study is provided in Table 1, together with information about the respective origin. Wine strains were isolated from fermenting musts in wine cellars from the Bairrada wine region and from vineyards of Bairrada and Vinho Verde wine regions, according to Valero *et al*. [44]. Commercial wine strains were kindly provided by *Adega Cooperativa da Bairrada*, Cantanhede, Portugal. Clinical isolates were a kind gift of Prof. Mick Tuite from the University of Kent, Canterbury, UK.

Yeast strains were cultivated in 5 ml YEPD (1% yeast extract, 2% peptone, 2% glucose), at 30°C, with 185 rpm agitation, until cell density reached 10^8^–10^9 ^cells/ml, harvested and washed three times with distilled water by centrifugation for 5 minutes at 3000 g, resuspended in 200 μl lysis buffer (2% Triton X-100, 1% SDS, 100 mM NaCl, 1 mM EDTA, 10 mM Tris pH 8.0), and stored at -80°C until used for DNA extraction.

### DNA isolation

DNA was isolated as described by Hoffman and Winston [74], with some adaptations. For DNA extraction, 200 μl of 25:24:1 phenol/chloroform/isoamyl alcohol were added to the cells resuspendend in lysis buffer (see above), together with 300 mg of acid-washed glass beads (~500 μm diameter). The mixture was vortexed for 10 minutes before the addition of 200 μl of TE buffer (10 mM Tris-HCl, 1 mM EDTA, pH 8.0). Following a 5 minute centrifugation at 14000 rpm (Eppendorf centrifuge 5415 R) for phase separation, the DNA in the aqueous phase was precipitated with 1 ml ethanol (96%, v/v) at room temperature, resuspended in 400 μl of TE containing 60 mg RNaseA (GE Healthcare), and incubated at 37°C for 6 hours. The DNA was precipitated with 10 μl of 4 M ammonium acetate and 1 ml of room temperature absolute ethanol, and resuspended in 400 μl of TE buffer. Proteins were removed by treating DNA samples with 10 μg Proteinase K (Roche) and incubating overnight at 50°C. Finally, the DNA was collected by precipitation with 20 μl of 3.0 M sodium acetate pH 5.2 and 500 μl of ethanol (96%, v/v) at -20°C, followed by incubation at -80°C for 1 hour. After centrifugation at 14000 rpm for 20 minutes at 4°C, the final DNA pellet was carefully washed with 500 μl of ethanol (70% v/v), left to dry at room temperature, and resuspended in 200 μl of TE buffer.

### Genotyping analysis

The PCR/RFLP analysis of the *MET2 *gene was performed as described by Antunovics *et al*. [75] and of *OPY1, KIN82, MET6, KEL2 *and *CYR1 *genes as described by González *et al. *[33,34]. PCR analysis of delta sequences was performed as described [76,77].

### Microarray production

For the production of *in-house *spotted DNA-microarrays, 6388 70 mer oligonucleotides targeting the ORFeome of *Saccharomyces cerevisiae *(OPERON Yeast AROS v1.1 collection, Qiagen) were spotted twice on CodeLink activated slides (GE Healthcare), according to the slide manufacturer's instructions, using a MicroGrid Compact II spotter (GenomicSolutions). A set of ten different 70 mer probes designed from *Escherichia coli *genome sequence, with less than 70% homology to *S. cerevisiae *genome, was also included in the microarray in order to monitor non-specific hybridization. The array design and spotting protocol were deposited in ArrayExpress database [78] under the accession code A-MEXP-1185.

### Labelling and hybridization

For labelling, 4 μg of gDNA were digested with 20 units of *Dpn*II (New England Biolabs, USA) to yield fragments between 250 and 3000 bp. The fragmented DNA was precipitated with 2.5 volumes of ethanol (96%, v/v) at -20°C. Genomic DNA was fluorescently labelled using the ULS arrayCGH labelling kit from Kreatech (Kreatech, The Netherlands), which is a non-enzymatic protocol that allows direct labelling of unmodified genomic DNA. Briefly, 1 μl of Cy3-ULS, or Cy5-ULS, was added to 2 μg of previously digested DNA, together with 2 μl of the 10 × labelling solution provided with the kit. The sample volume was adjusted to 20 μl with DNAse-free water and the labelling reaction was promoted by incubating the sample at 85°C for 30 minutes. The excess ULS-dye was removed using the KREA *pure *columns following the kit manufacturer's instructions. The degree of labelling (DoL), corresponding to the percentage of labelled nucleotides, was determined by measuring the absorbance at 260 nm and at 550 nm for ULS-Cy3 labelled DNA, or at 260 nm and 650 nm for ULS-Cy5 labelled DNA. Samples with DoL between 1.0% and 2.0% were routinely obtained.

For comparative genome hybridization, ULS-Cy3 labelled DNA from each of the environmental, commercial and clinical strains was combined with ULS-Cy5 labelled DNA from strain S288C. Dye-swap hybridizations were performed for each strain. To ensure microarray data baseline robustness, differentially labelled DNA from the S288C strain were co-hybridized, in a total of six self-self experiments, and used as controls. The mixture of the required Cy3 and Cy5 labelled samples was adjusted to 208 μl with DNAse-free water, mixed with 52 μl Agilent 10× blocking Agent and 260 μl of Agilent 2× Oligo aCGH Hybridization Solution (Agilent, USA), and incubated at 95°C for 3 minutes. After a short spin-down, the labelled DNA mixture was applied to a microarray slide pre-hybridized as described by van de Peppel and colleagues [79], assembled in a SureHyb hybridization chamber fitted with a gasket slide (Agilent), and incubated for 20 hours at 65°C in a hybridization oven (HIR10M Grant, Boekel), with 5 rpm rotation speed.

Slides were washed as described in the Agilent Oligonucleotide Array-based CGH for Genomic DNA Analysis protocol [80]. Briefly, the microarray and gasket slide were disassembled inside a staining dish containing 250 ml of Oligo aCGH Wash Buffer 1 and the slides (up to 4) were washed in fresh 250 ml of Oligo aCGH Wash Buffer 1 solution at room temperature, during 5 minutes, with gentle agitation from a magnetic stirrer. A second wash step was carried out by immersing the slides in Oligo aCGH Wash Buffer 2 solution, previously warmed to 37°C, during 1 minute, also with gentle magnetic stirring. Finally, slides were dried by centrifugation at 800 rpm for 3 minutes.

### Image acquisition and data processing

Images of the microarray hybridizations were acquired using the Agilent G2565AA microarray scanner. The fluorescence intensities were quantified with QuantArray v3.0 software (PerkinElmer). Using BRB-ArrayTools v3.4.0 software [81], manually flagged bad spots were eliminated and the local background was subtracted before averaging the replicate features on the array. Log_2 _intensity ratios (M values) were then Median normalized to correct for differences in genomic DNA labelling efficiency between samples. The raw data, as well as the processed (filtered and Median normalized) data, for all hybridizations was submitted to the ArrayExpress database and is available under the accession code E-MTAB-29.

### Data analysis

The relative hybridization signal of each ORF was derived form the average of the two dye-swap hybridizations performed for each strain. The normalized log_2 _ratio (M value) was considered as a measure of the relative abundance of each ORF relatively to that of the reference strain S288C. Deviations from the 1:1 hybridization ratio were taken as indicative of changes in DNA copy number. Although depleted hybridization may be due to sequence divergence as well as nucleotide deletions, sequence divergence of a given ORF relatively to that of S288C would have to be higher than 30% in order to impair the hybridization with 70 mer oligonucleotides [82,83]. Given that the variability usually observed between *Saccharomyces *genomes, (either within laboratorial strains or natural isolates), is much lower than this estimate [11,32,41], we interpreted statistically significant depletions in hybridization signal as ORF deletions. We further confirmed that the set of ORFs discussed in Figure 6 were targeted by the respective microarray probe with at least 93% homology in the genomes of the *S. cerevisiae *strains S288C, RM11-1a (wine isolate) and YJM789 (clinical isolate). This excluded the hypothesis of depleted hybridization signal due to extreme gene variability or partial deletion of the targeted sequence (Additional File 4, Table S1).

The aCGH hybridization patterns were used to investigate the genomic relatedness of the wild-type yeast strains. Hierarchical cluster analysis (Pearson correlation, average linkage) was performed using MeV from TM4 software suit [84], with the normalized and dye-swap averaged aCGH profiles. The reproducibility of the clustering analysis was confirmed by repeating the paired dye-swap hybridizations for one of the sixteen strains, randomly selected, and by verifying that independent assays for the same strain grouped together in the dendrogram (not shown).

Karyoscope maps were generated for each strain using CGH-Miner [85]. For data smoothing, the parameters were set for BAC analysis, to produce a moving window of three ORFs for averaging the hybridization signal. Six S288C independent self-self hybridizations were used for base line correction. The average data of the six self-self S288C hybridizations was used to ascertain the baseline noise in deriving karyoscope maps and is shown in Additional File 2, Figure S2Q. Summary plots, also denominated consensus plots, depicting the relative percentage of samples showing a particular alteration, were obtained by combining the required individual karyoscope maps using the same software.

Multi-class significance analysis (SAM) was done using the algorithm implemented in MeV from TM4 software. The individual hybridizations were used as the input data, in a total of two dye-swap hybridizations for each strain/class, except for strain S288C, which was represented by the five least variable self-self hybridizations of the set of six performed for CLAC analysis. SAM analysis indicated the ORFs with significant copy number alteration in at least one of the strains, with a FDR (90^th ^percentile) of 0.336. Functional annotations and GO terms association was done following the *Saccharomyces *Genome Database (SGD) annotations [86].

## Abbreviations

aCGH: array Comparative Genome Hybridization; FDR: False Discovery Rate; SAM: Significance Analysis of Microarrays; SGD: Saccharomyces Genome Database; SNP: Single Nucleotide Polimorphism

## Competing interests

The authors declare that they have no competing interests.

## Authors' contributions

LC performed experiments, helped in the experimental design, supervised the experiments, performed data analysis and wrote the manuscript. MFE performed the experiments and data analysis. ACG isolated, identified and genotyped the environmental Bairrada wine yeast strains, PP contributed to data analysis, DS participated in the design of the study, provided the Vinho Verde strains and performed the simple sequence repeats analysis. MAS coordinated the study, wrote and proofread the manuscript. All authors read and approved the final manuscript.

## Supplementary Material

Additional File 1 **Hybrid detection by PCR-RFLP analysis.** PCR-RFLP of five distinct loci located in different chromosomes. Click here for file

Additional File 2 **CGH Miner karyoscope maps.** Karyoscope maps of the 16 wild-type strains analyzed (S2A-S2P) and the baseline karyoscope obtained for strain S288C (S2Q). Click here for file

Additional File 3 **Genome alterations between YJM789 and S288C and this study.** Genome alterations found in the consensus plot obtained for the wine strains are compared of the clinical strain YJM789 whose genome is fully sequenced. Click here for file

Additional File 4 **Confirmation of microarray probe targets for a selected group of ORF.** Specificity and homology of a set of microarray probes using BLAST alignment against the genomes of *S. cerevisiae *strains S288C, RM11-1a and YJM789. Click here for file
