# Supplementary material for: Comparative genomics of wild type yeast strains unveils important genome diversity
Source: BMC Genomics. 2008 Nov 4;9:524. doi: 10.1186/1471-2164-9-524 (PMC2588607; doi:10.1186/1471-2164-9-524)
Supplement: Additional File 1 — Hybrid detection by PCR-RFLP analysis. PCR-RFLP of five distinct loci located in different chromosomes. [file 1471-2164-9-524-S1.pdf]

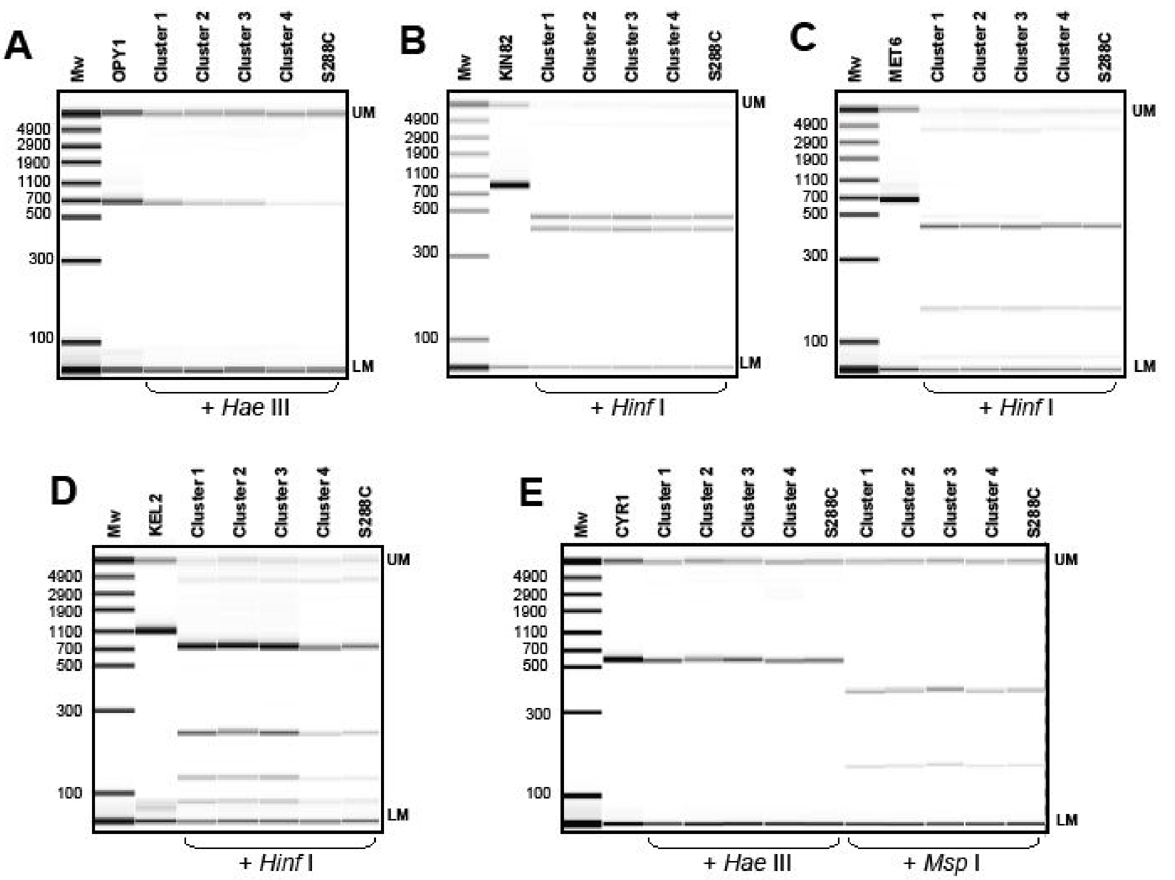

Figure S1

***Saccharomyces* hybrid screen by PCR-RFLP analysis.**

The strains used in this study were tested for their hybrid nature by PCR-RFLP of five distinct loci located in different chromosomes. A) RFLP of the *OPY1* locus, in chromosome II, with the *Hae* III; B) RFLP of the *KIN82* locus, in chromosome III, with the *Hinf* I; C) RFLP of the *MET6* locus, in chromosome V, with the *Hinf* I; D) RFLP of the *KEL2* locus, in chromosome VII, with the *Hinf* I; and E) RFLP of the *KCYR1* locus, in chromosome X, with both *Hae* III and *Msp* I. The strains have a *S. cerevisiae* restriction profile, thus ruling out the hypothesis of the presence of hybrids. We have performed this analysis on all the strains used in this study. The panels above show only selected profiles of strains 06L3FF02, J940047, Lalvin ICV D254 and UM237 which are representative of Clusters 1, 2, 3 and 4 from Figure 2 of the manuscript, respectively. Strain S288C was used as a control for *S. cerevisiae*-specific RFLP patterns. All PCR amplifications and restriction reactions were done as described in the Methods section. The fragment analysis was carried out by DNA electrophoresis on a Labchip HT (Caliper LS), and the data was displayed using the DataViewer software (Caliper LS). Each lane is identified at the top. UM – Upper marker; LM – Lower marker.
