# Supplementary material for: Comparative genomics of wild type yeast strains unveils important genome diversity
Source: BMC Genomics. 2008 Nov 4;9:524. doi: 10.1186/1471-2164-9-524 (PMC2588607; doi:10.1186/1471-2164-9-524)
Supplement: Additional File 2 — CGH Miner karyoscope maps. Karyoscope maps of the 16 wild-type strains analyzed (S2A-S2P) and the baseline karyoscope obtained for strain S288C (S2Q). [file 1471-2164-9-524-S2.pdf]

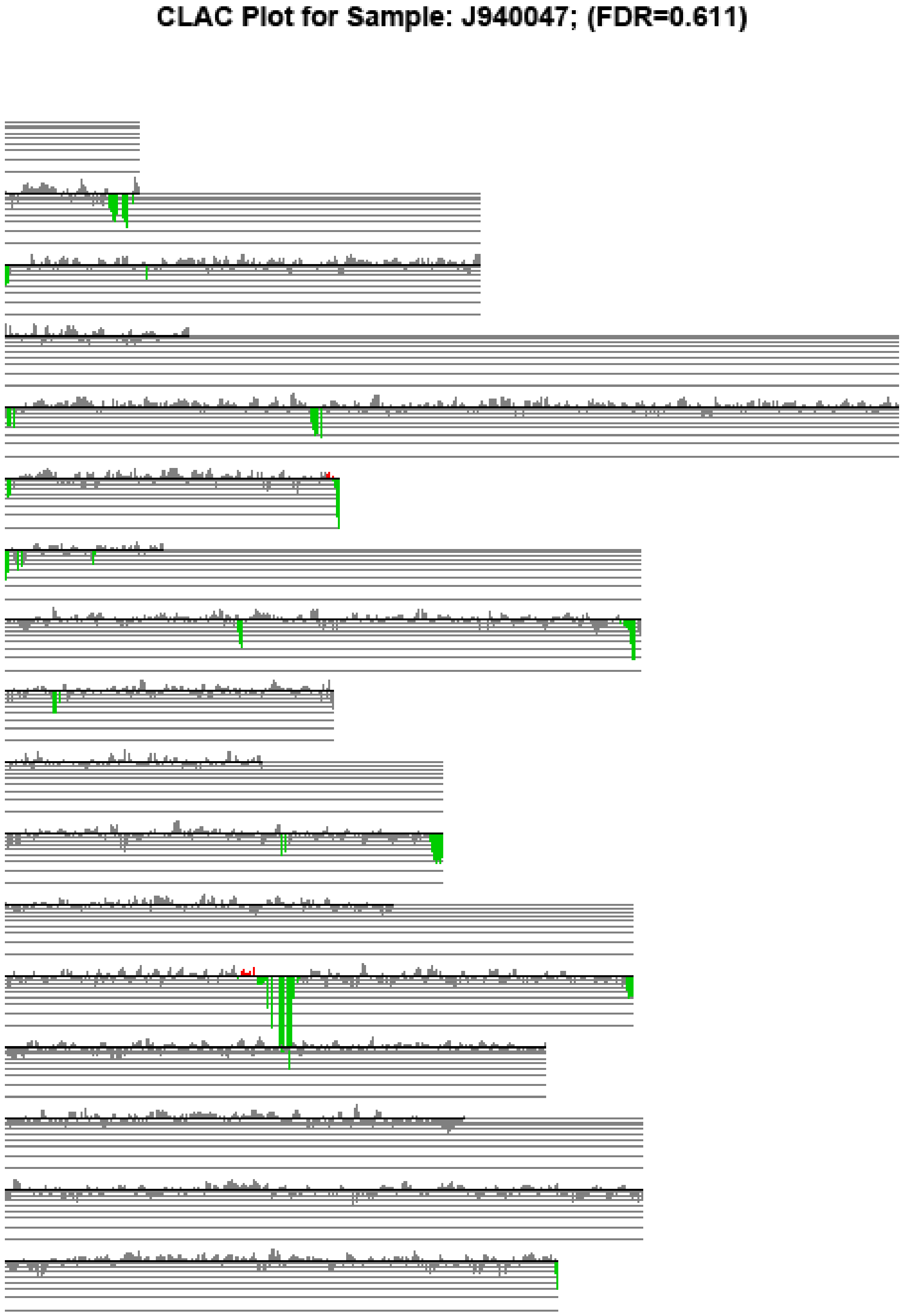

Figure S2A

Karyoscope maps for strain J940047.

For the identification of clusters of ORFs with copy number alterations, the aCGH data was displayed along the chromosomes, using the annotated ORF coordinates of S288C. Vertical bars represent the relative hybridization pattern relatively to the genome of strain S288C. Red bars correspond to amplified ORFs, green bars represent deleted ORFs and grey bars are statistically non-significant alterations. The horizontal lines indicate the hybridization ratios in logarithmic scale. The map was obtained with CGH-Miner, using an averaging moving window of three ORFs.

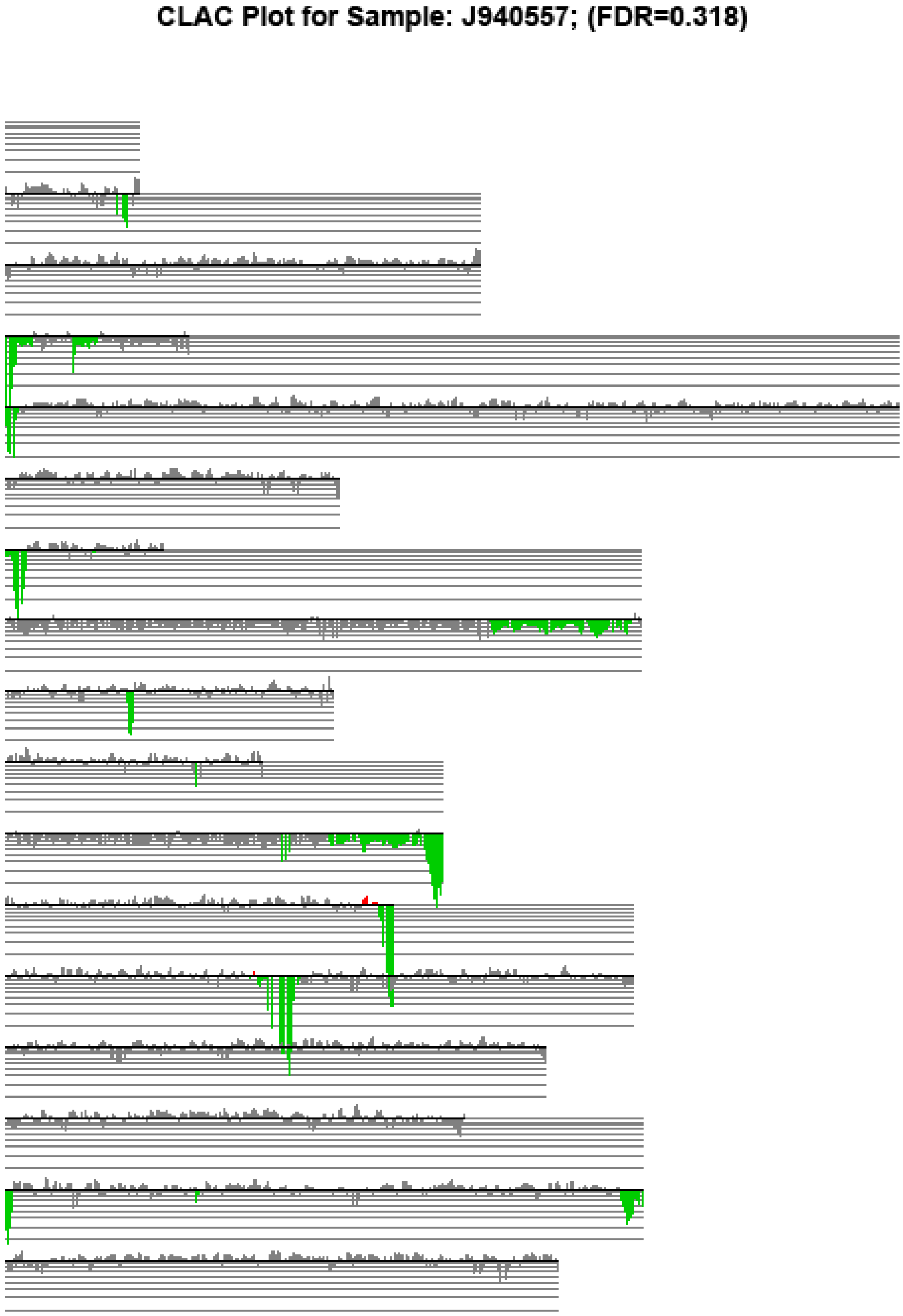

Figure S2B
Karyoscope maps for strain J940557.
For the identification of clusters of ORFs with copy number alterations, the aCGH data was displayed along the chromosomes, using the annotated ORF coordinates of S288C. Vertical bars represent the relative hybridization pattern relatively to the genome of strain S288C. Red bars correspond to amplified ORFs, green bars represent deleted ORFs and grey bars are statistically non-significant alterations. The horizontal lines indicate the hybridization ratios in logarithmic scale. The map was obtained with CGH-Miner, using an averaging moving window of three ORFs.

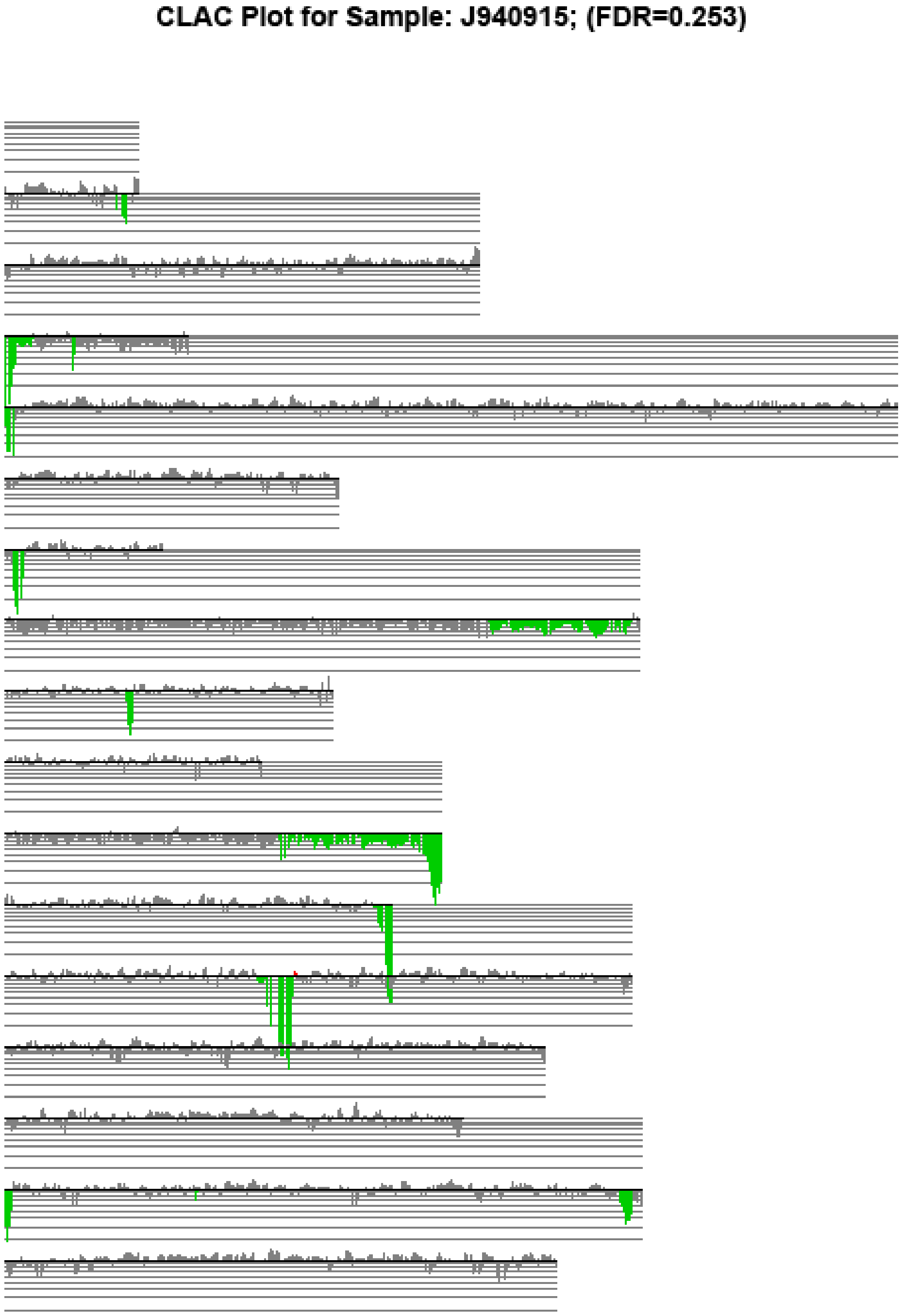

Figure S2C
Karyoscope maps for strain J940915.
For the identification of clusters of ORFs with copy number alterations, the aCGH data was displayed along the chromosomes, using the annotated ORF coordinates of S288C. Vertical bars represent the relative hybridization pattern relatively to the genome of strain S288C. Red bars correspond to amplified ORFs, green bars represent deleted ORFs and grey bars are statistically non-significant alterations. The horizontal lines indicate the hybridization ratios in logarithmic scale. The map was obtained with CGH-Miner, using an averaging moving window of three ORFs.

CLAC Plot for Sample: Lalvin EC−1118; (FDR=0.393)

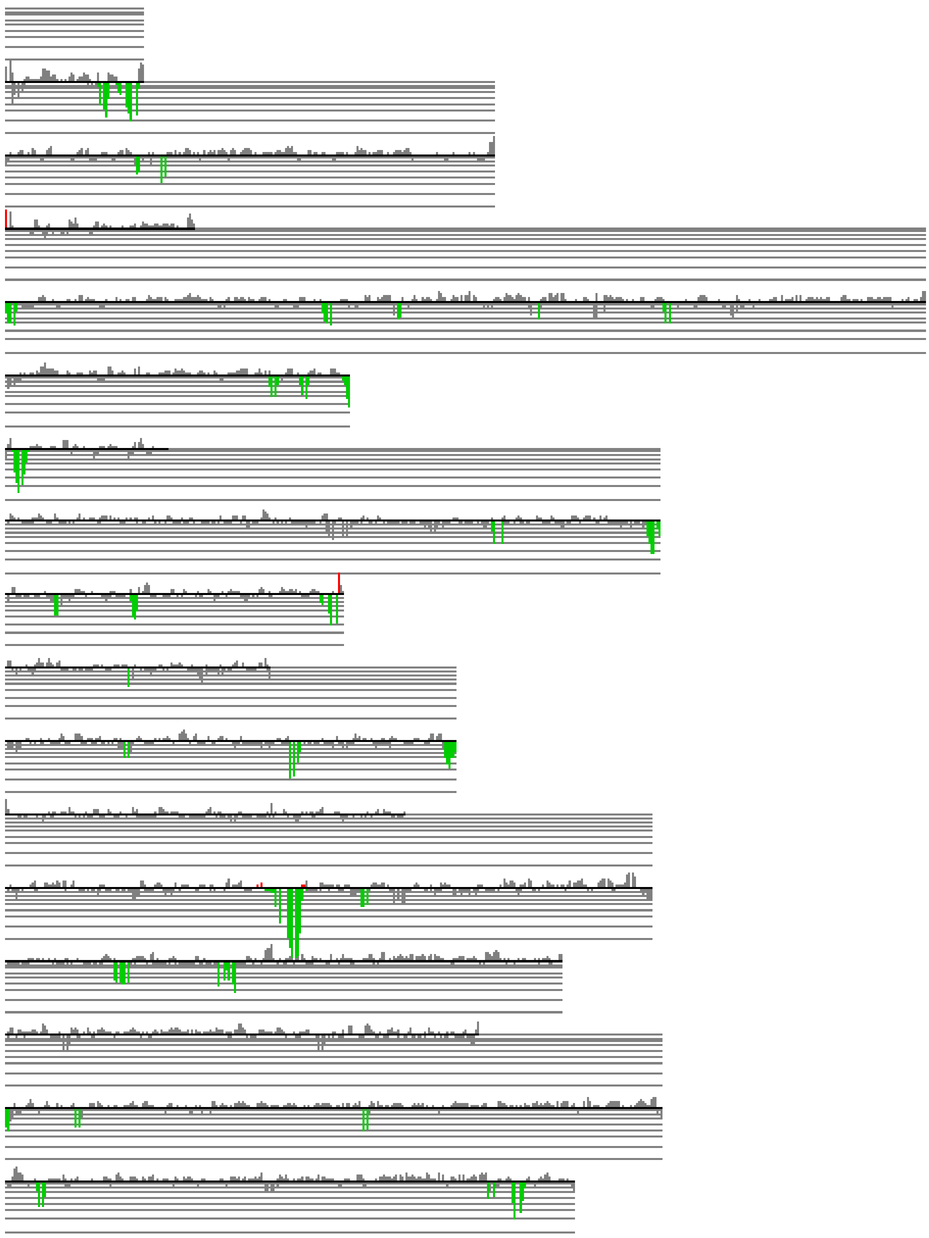

Figure S2D

Karyoscope maps for strain Lalvin EC-1118.

For the identification of clusters of ORFs with copy number alterations, the aCGH data was displayed along the chromosomes, using the annotated ORF coordinates of S288C. Vertical bars represent the relative hybridization pattern relatively to the genome of strain S288C. Red bars correspond to amplified ORFs, green bars represent deleted ORFs and grey bars are statistically non-significant alterations. The horizontal lines indicate the hybridization ratios in logarithmic scale. The map was obtained with CGH-Miner, using an averaging moving window of three ORFs.

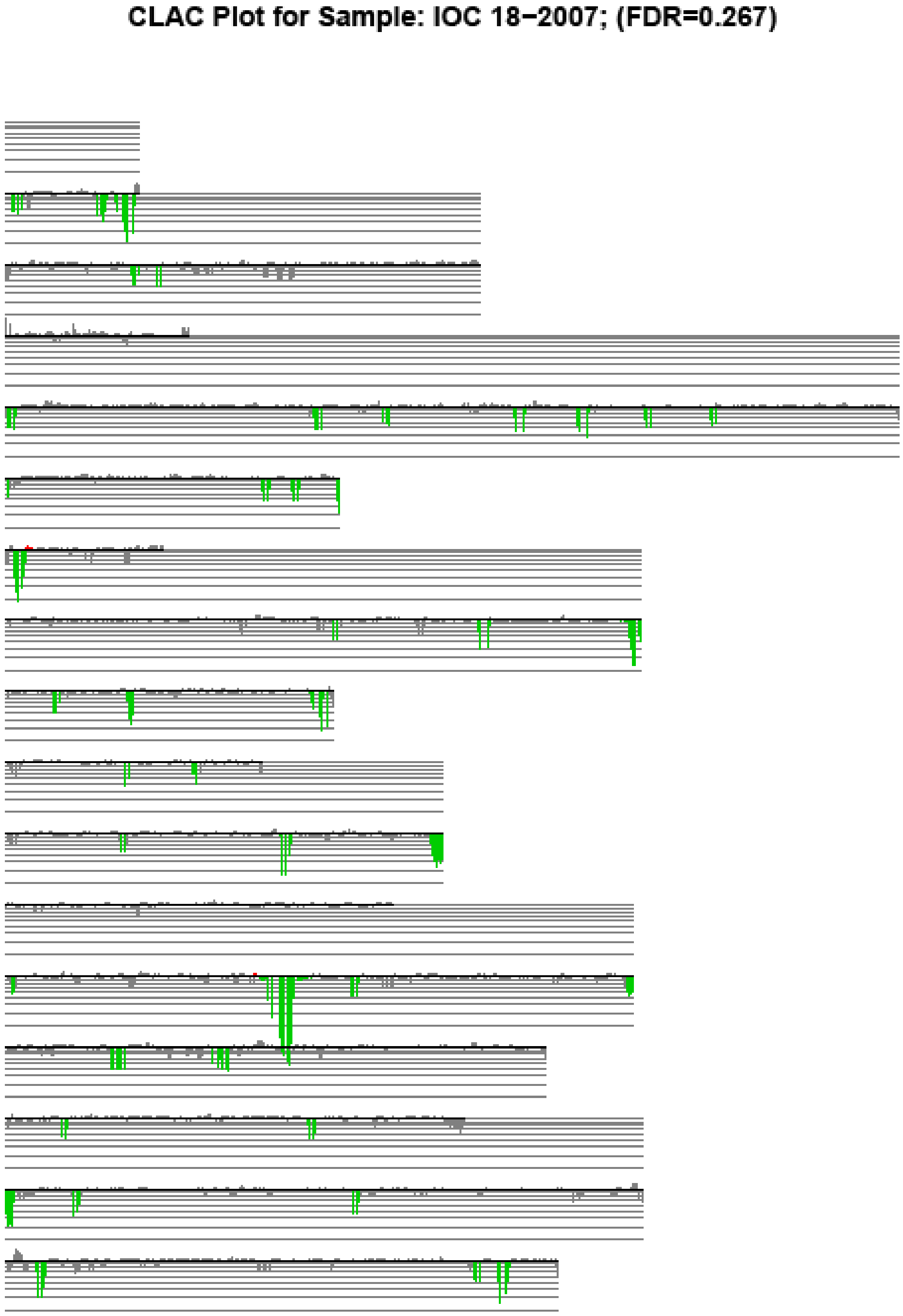

Figure S2E

Karyoscope maps for strain IOC 18-2007.

For the identification of clusters of ORFs with copy number alterations, the aCGH data was displayed along the chromosomes, using the annotated ORF coordinates of S288C. Vertical bars represent the relative hybridization pattern relatively to the genome of strain S288C. Red bars correspond to amplified ORFs, green bars represent deleted ORFs and grey bars are statistically non-significant alterations. The horizontal lines indicate the hybridization ratios in logarithmic scale. The map was obtained with CGH-Miner, using an averaging moving window of three ORFs.

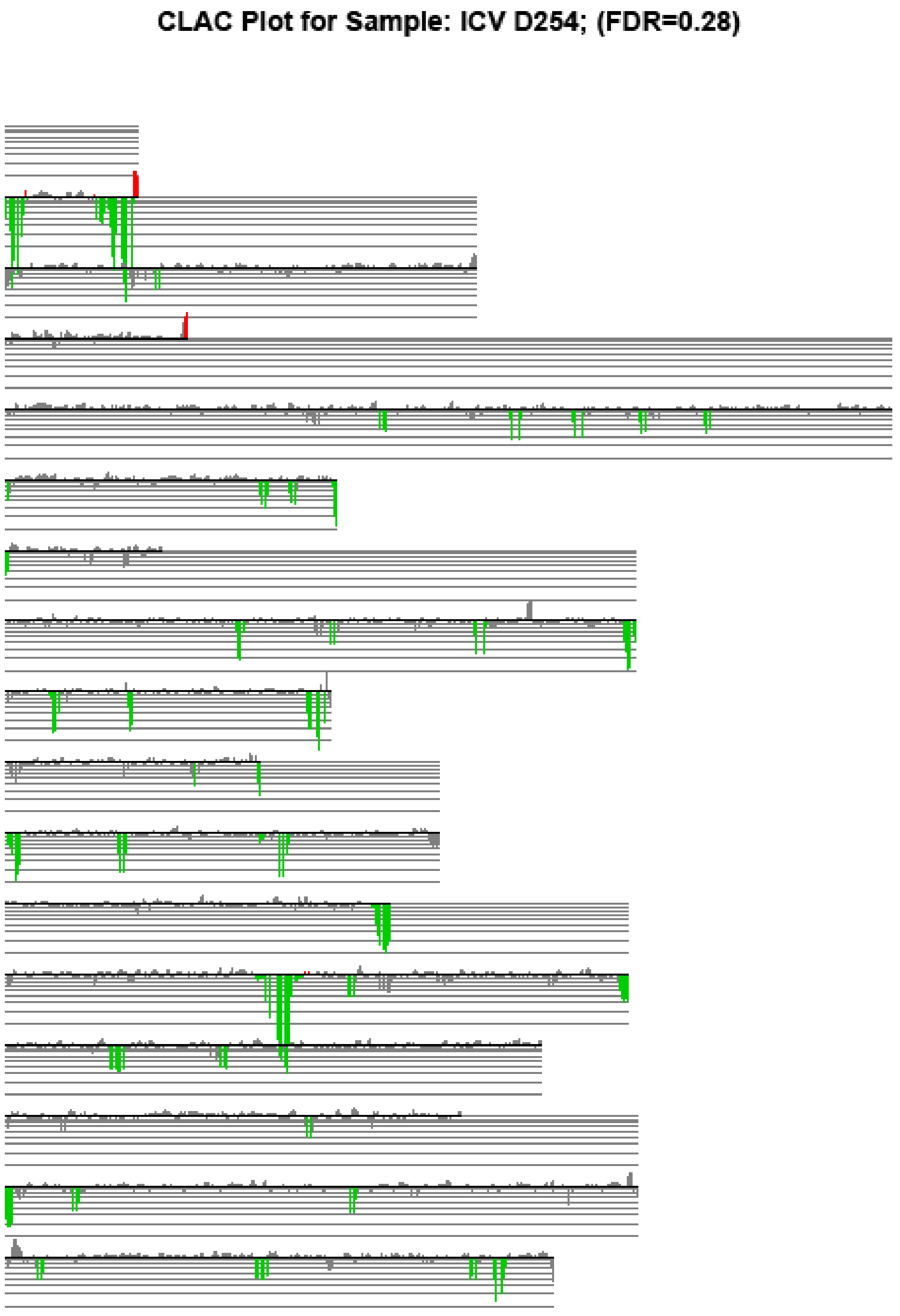

Figure S2F
Karyoscope maps for strain Lalvin ICV D254.
For the identification of clusters of ORFs with copy number alterations, the aCGH data was displayed along the chromosomes, using the annotated ORF coordinates of S288C. Vertical bars represent the relative hybridization pattern relatively to the genome of strain S288C. Red bars correspond to amplified ORFs, green bars represent deleted ORFs and grey bars are statistically non-significant alterations. The horizontal lines indicate the hybridization ratios in logarithmic scale. The map was obtained with CGH-Miner, using an averaging moving window of three ORFs.

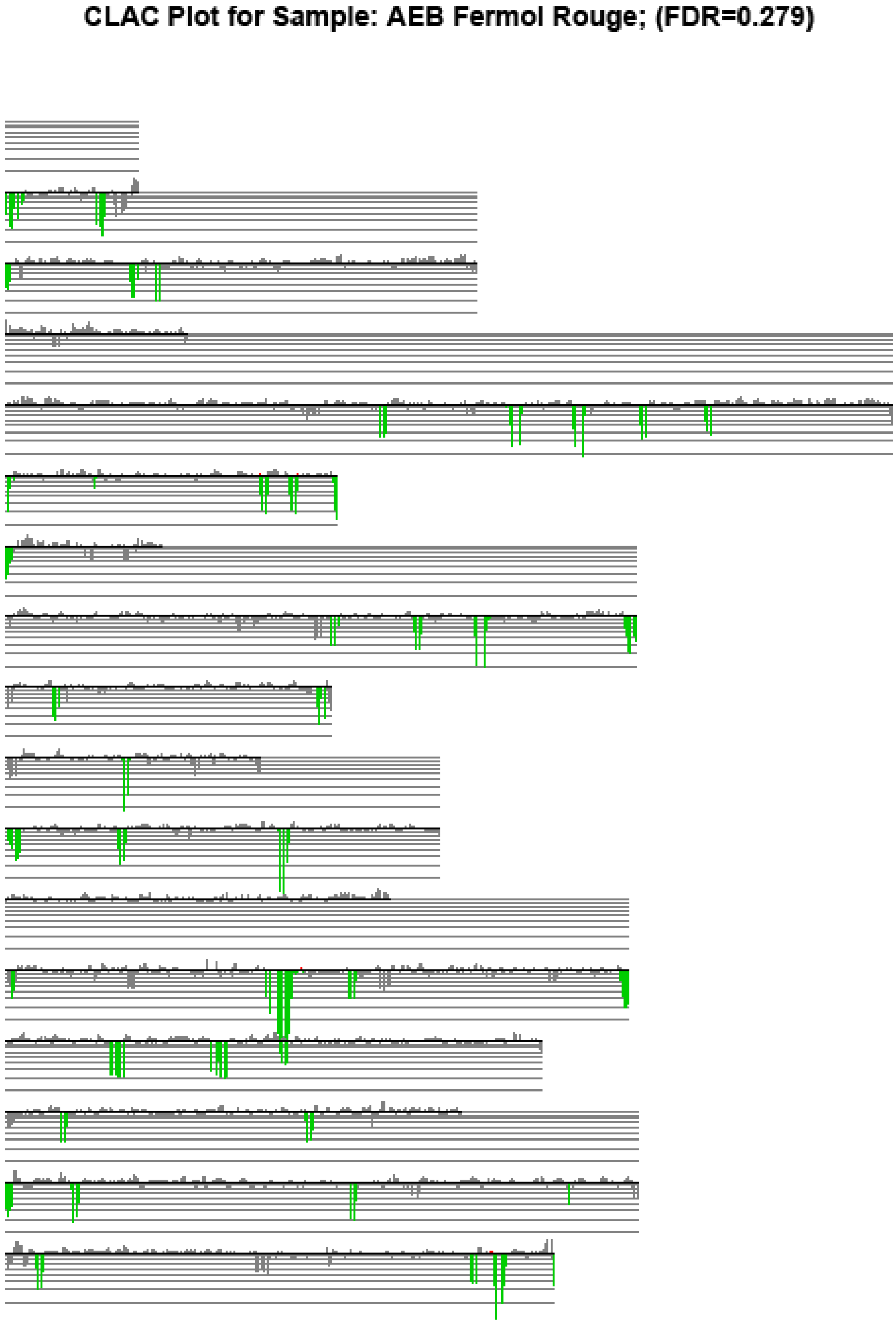

Figure S2G

Karyoscope maps for strain AEB Fermol Rouge.

For the identification of clusters of ORFs with copy number alterations, the aCGH data was displayed along the chromosomes, using the annotated ORF coordinates of S288C. Vertical bars represent the relative hybridization pattern relatively to the genome of strain S288C. Red bars correspond to amplified ORFs, green bars represent deleted ORFs and grey bars are statistically non-significant alterations. The horizontal lines indicate the hybridization ratios in logarithmic scale. The map was obtained with CGH-Miner, using an averaging moving window of three ORFs.

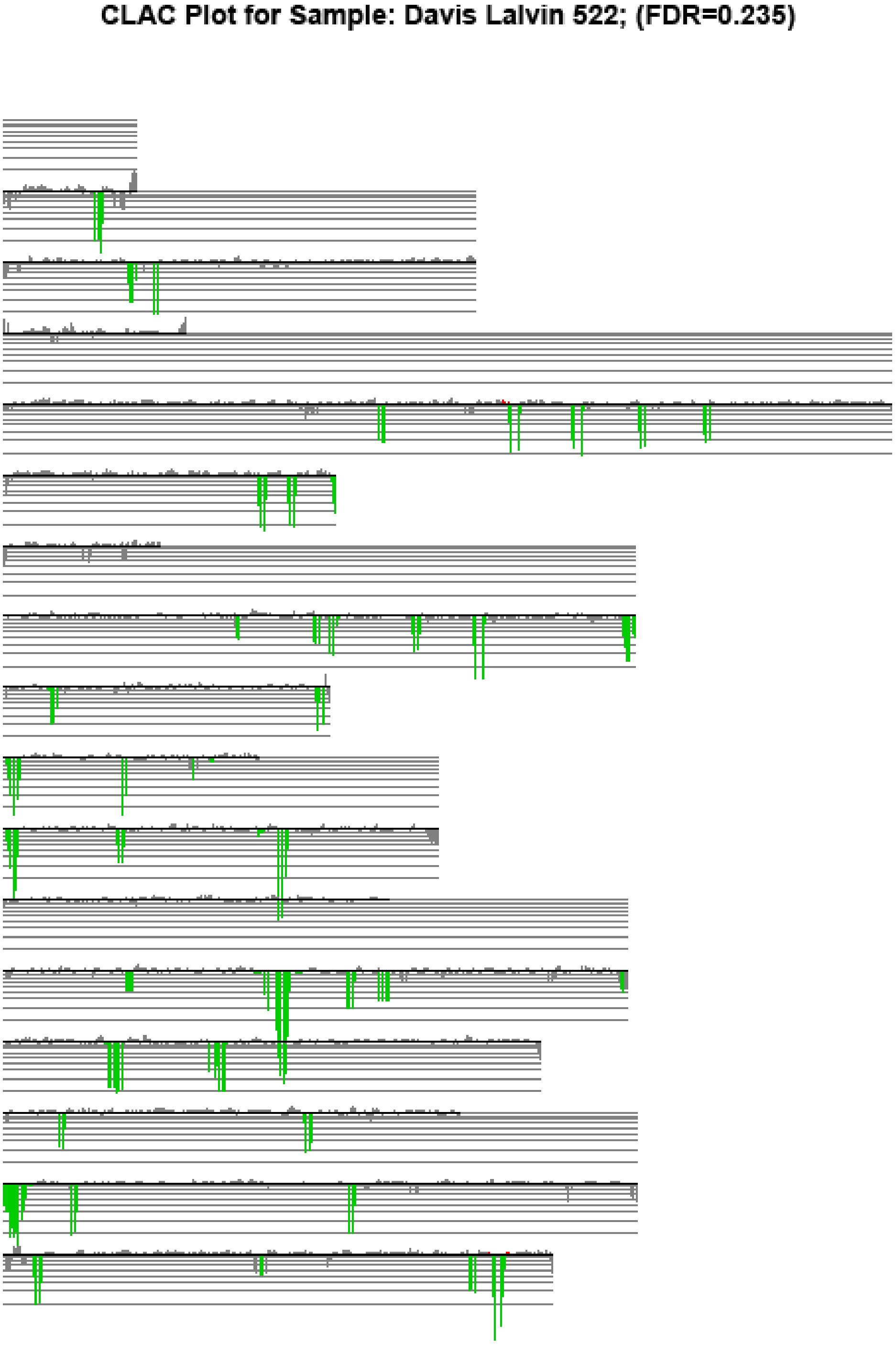

Figure S2H

Karyoscope maps for strain Davis Lalvin 522.

For the identification of clusters of ORFs with copy number alterations, the aCGH data was displayed along the chromosomes, using the annotated ORF coordinates of S288C. Vertical bars represent the relative hybridization pattern relatively to the genome of strain S288C. Red bars correspond to amplified ORFs, green bars represent deleted ORFs and grey bars are statistically non-significant alterations. The horizontal lines indicate the hybridization ratios in logarithmic scale. The map was obtained with CGH-Miner, using an averaging moving window of three ORFs.

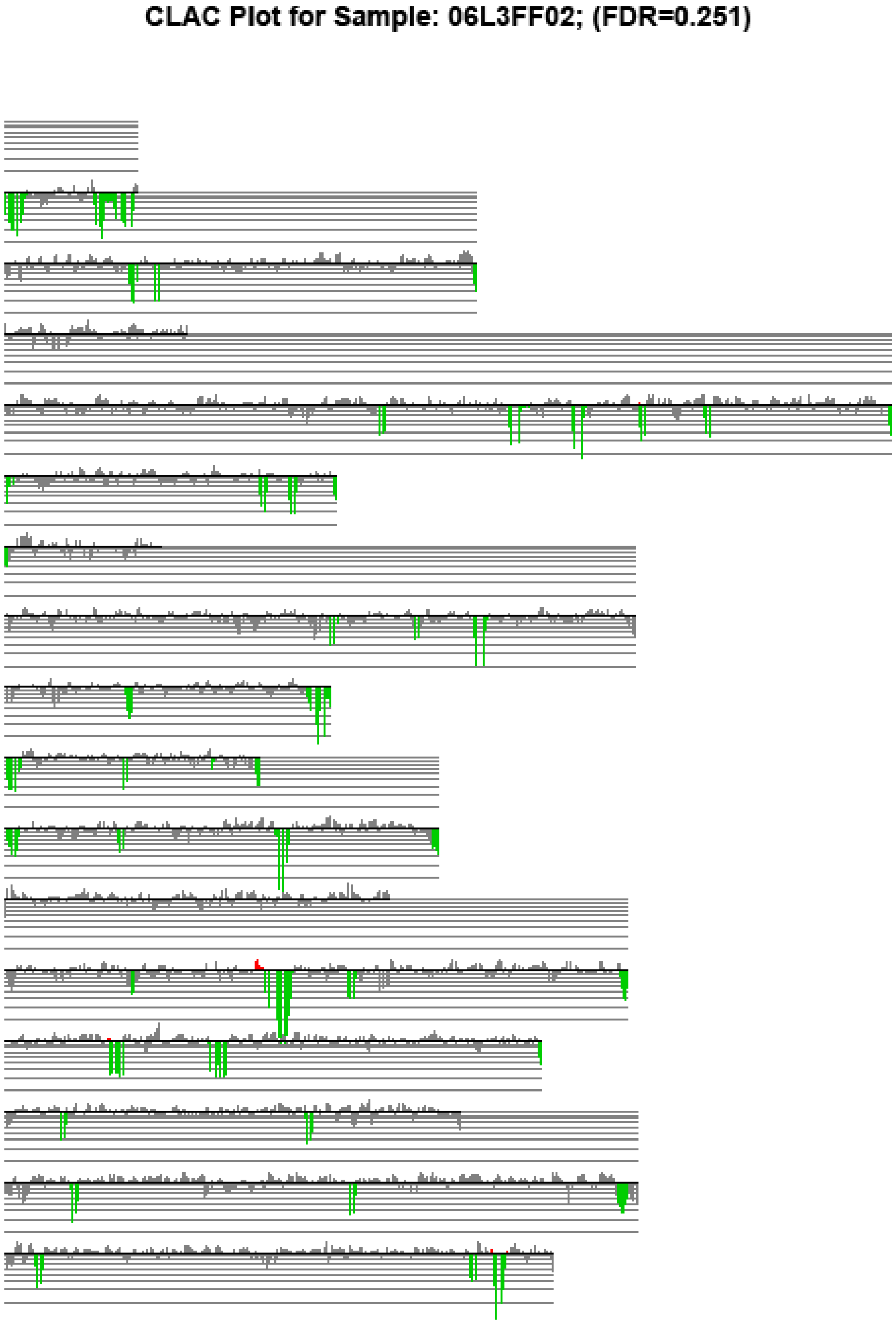

Figure S2I

Karyoscope maps for strain 06L3FF02.

For the identification of clusters of ORFs with copy number alterations, the aCGH data was displayed along the chromosomes, using the annotated ORF coordinates of S288C. Vertical bars represent the relative hybridization pattern relatively to the genome of strain S288C. Red bars correspond to amplified ORFs, green bars represent deleted ORFs and grey bars are statistically non-significant alterations. The horizontal lines indicate the hybridization ratios in logarithmic scale. The map was obtained with CGH-Miner, using an averaging moving window of three ORFs.

CLAC Plot for Sample: 06L1FF11; (FDR=0.206)

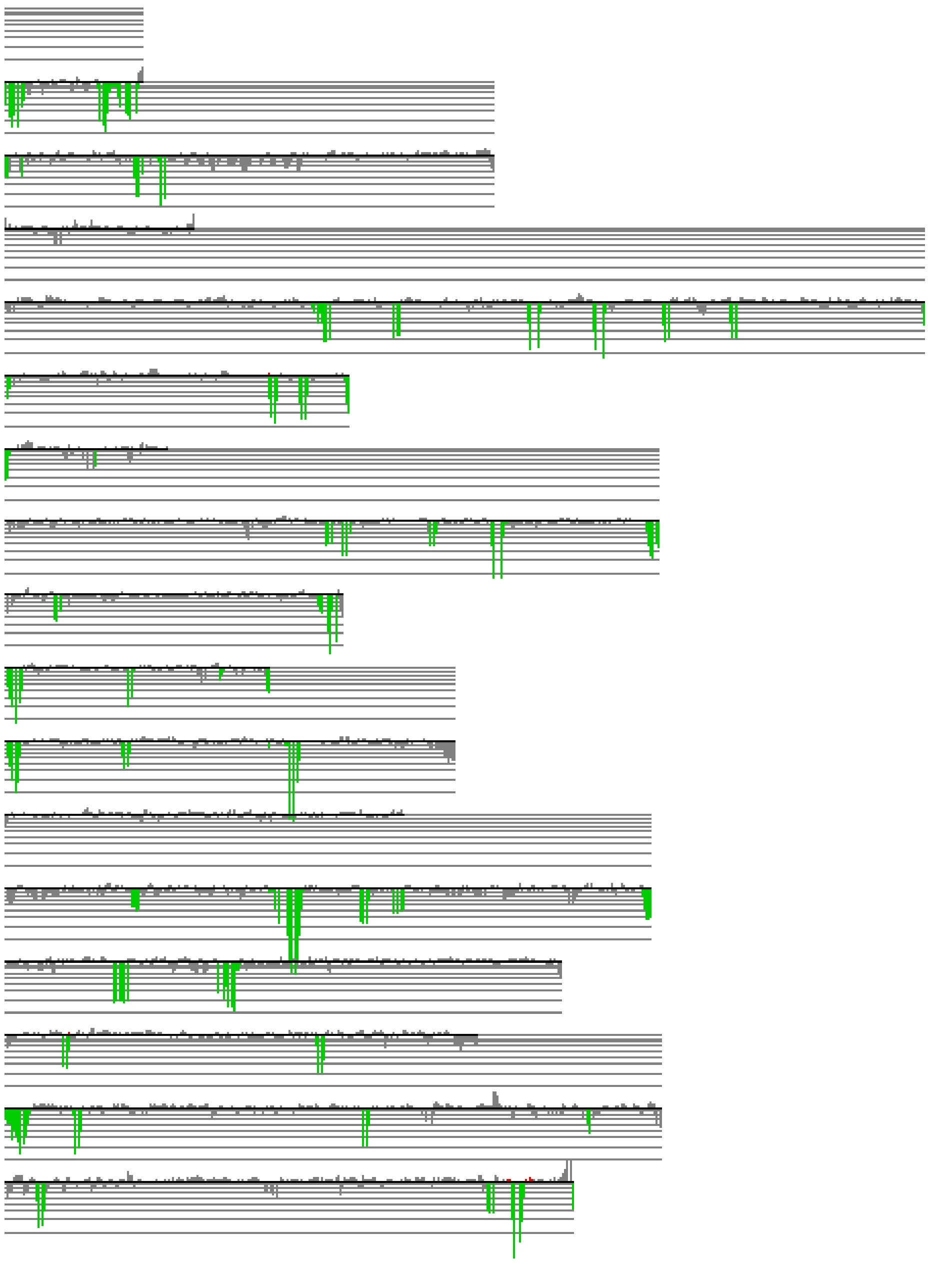

Figure S2J

Karyoscope maps for strain 06L1FF11.

For the identification of clusters of ORFs with copy number alterations, the aCGH data was displayed along the chromosomes, using the annotated ORF coordinates of S288C. Vertical bars represent the relative hybridization pattern relatively to the genome of strain S288C. Red bars correspond to amplified ORFs, green bars represent deleted ORFs and grey bars are statistically non-significant alterations. The horizontal lines indicate the hybridization ratios in logarithmic scale. The map was obtained with CGH-Miner, using an averaging moving window of three ORFs.

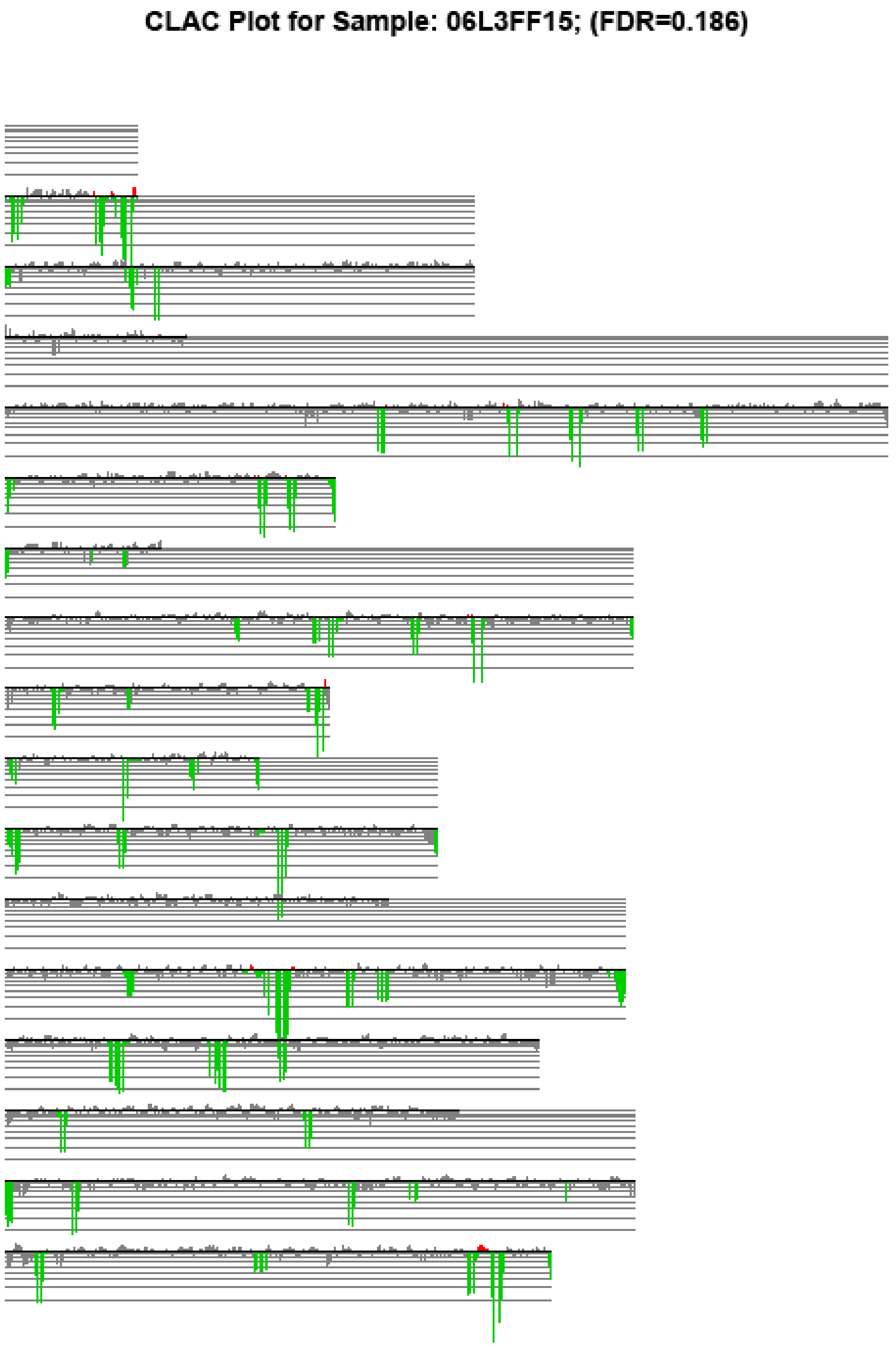

Figure S2K
Karyoscope maps for strain 06L3FF15.
For the identification of clusters of ORFs with copy number alterations, the aCGH data was displayed along the chromosomes, using the annotated ORF coordinates of S288C. Vertical bars represent the relative hybridization pattern relatively to the genome of strain S288C. Red bars correspond to amplified ORFs, green bars represent deleted ORFs and grey bars are statistically non-significant alterations. The horizontal lines indicate the hybridization ratios in logarithmic scale. The map was obtained with CGH-Miner, using an averaging moving window of three ORFs.

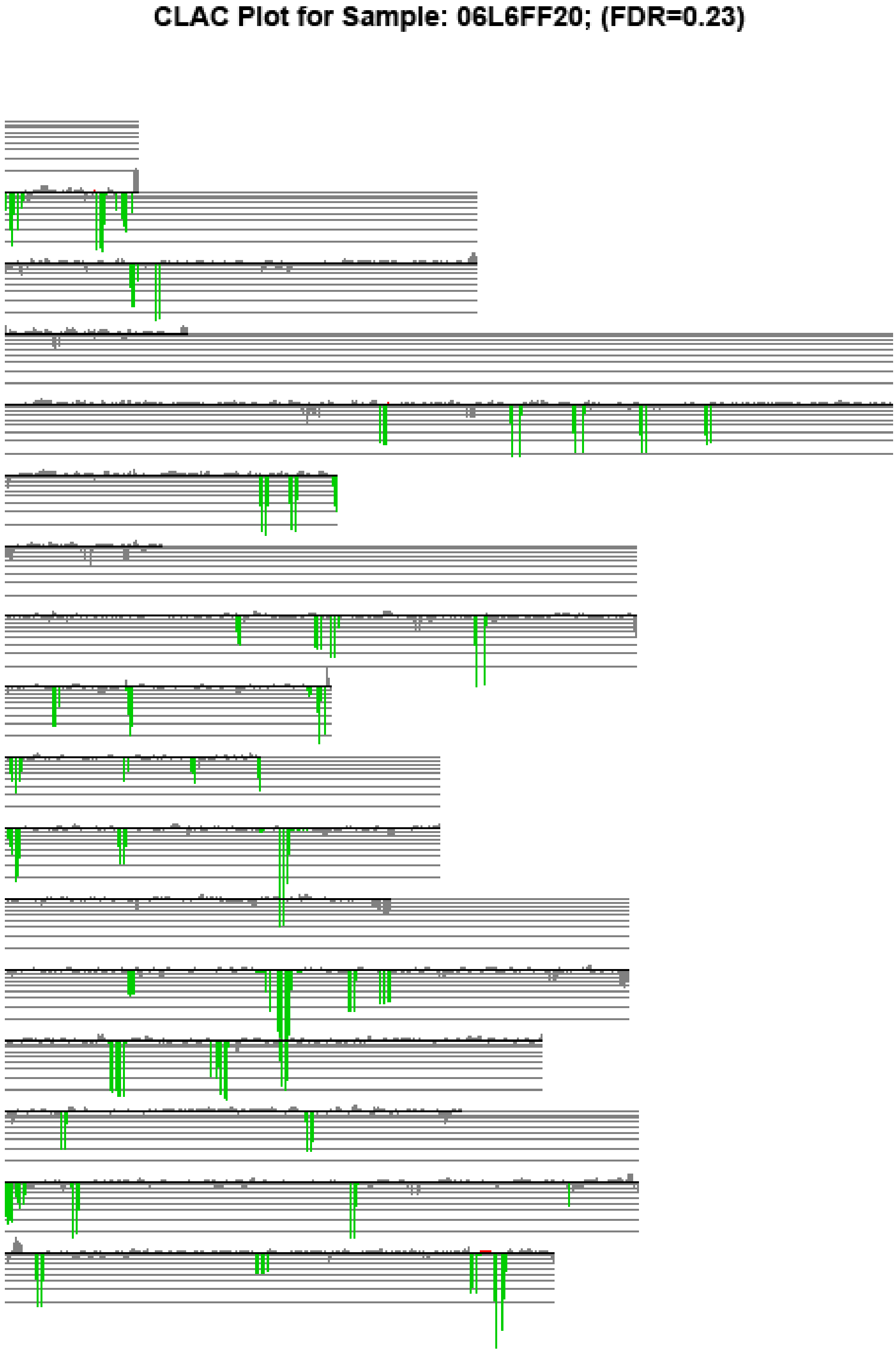

Figure S2L
Karyoscope maps for strain 06L6FF20.
For the identification of clusters of ORFs with copy number alterations, the aCGH data was displayed along the chromosomes, using the annotated ORF coordinates of S288C. Vertical bars represent the relative hybridization pattern relatively to the genome of strain S288C. Red bars correspond to amplified ORFs, green bars represent deleted ORFs and grey bars are statistically non-significant alterations. The horizontal lines indicate the hybridization ratios in logarithmic scale. The map was obtained with CGH-Miner, using an averaging moving window of three ORFs.

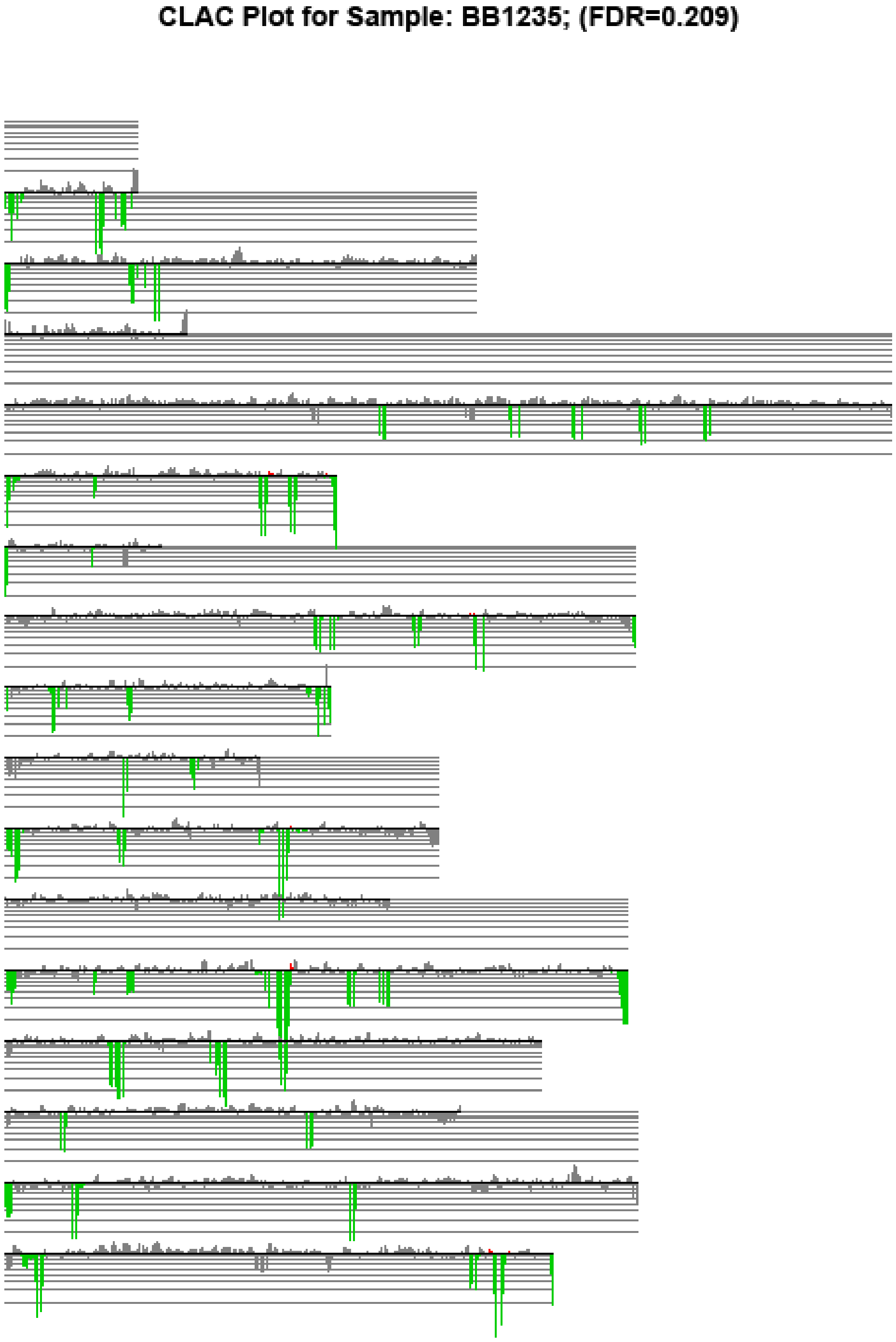

Figure S2M

Karyoscope maps for strain BB1235.

For the identification of clusters of ORFs with copy number alterations, the aCGH data was displayed along the chromosomes, using the annotated ORF coordinates of S288C. Vertical bars represent the relative hybridization pattern relatively to the genome of strain S288C. Red bars correspond to amplified ORFs, green bars represent deleted ORFs and grey bars are statistically non-significant alterations. The horizontal lines indicate the hybridization ratios in logarithmic scale. The map was obtained with CGH-Miner, using an averaging moving window of three ORFs.

CLAC Plot for Sample: BB2453; (FDR=0.275)

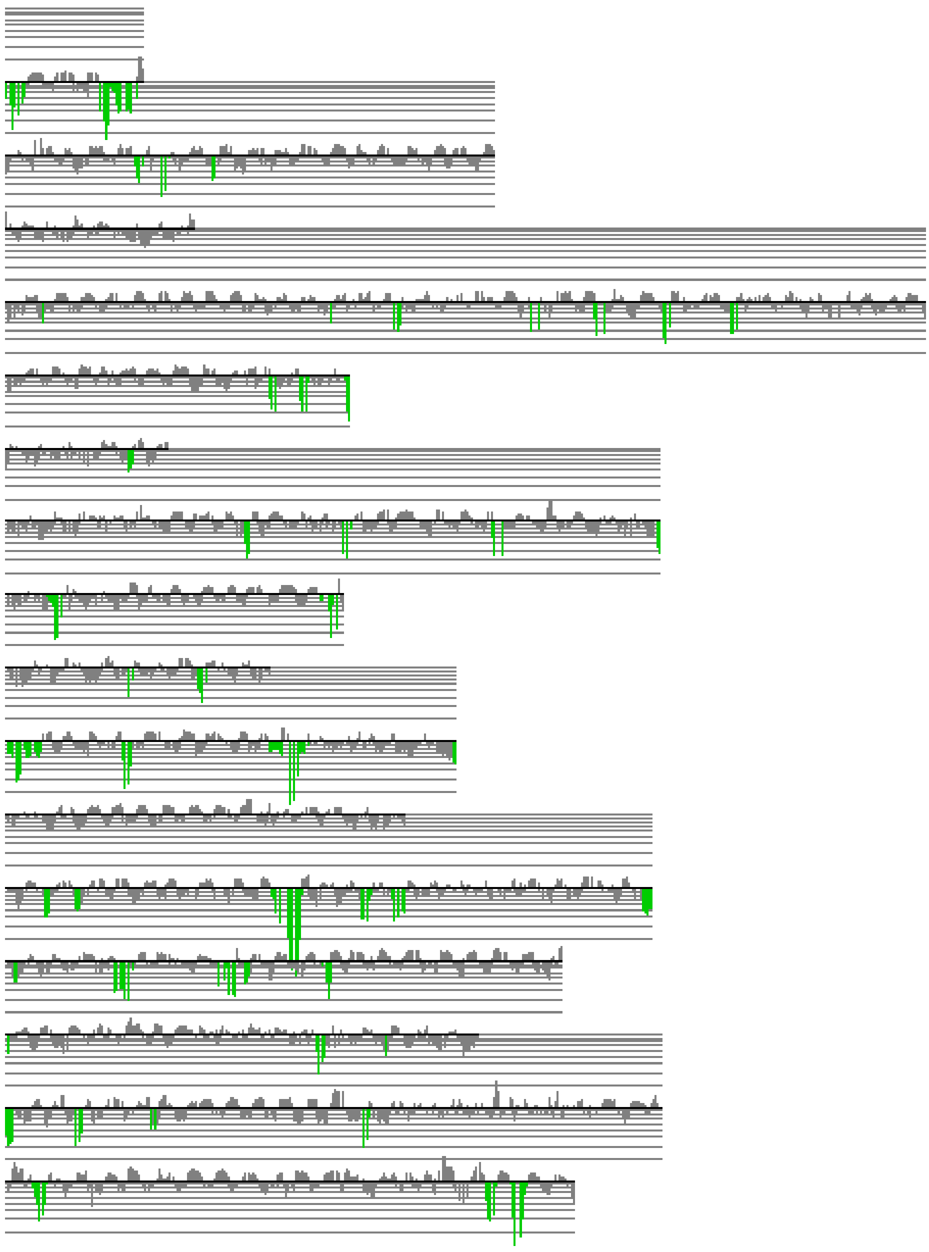

Figure S2N
Karyoscope maps for strain BB2453.
For the identification of clusters of ORFs with copy number alterations, the aCGH data was displayed along the chromosomes, using the annotated ORF coordinates of S288C. Vertical bars represent the relative hybridization pattern relatively to the genome of strain S288C. Red bars correspond to amplified ORFs, green bars represent deleted ORFs and grey bars are statistically non-significant alterations. The horizontal lines indicate the hybridization ratios in logarithmic scale. The map was obtained with CGH-Miner, using an averaging moving window of three ORFs.

CLAC Plot for Sample: UM218; (FDR=0.247)

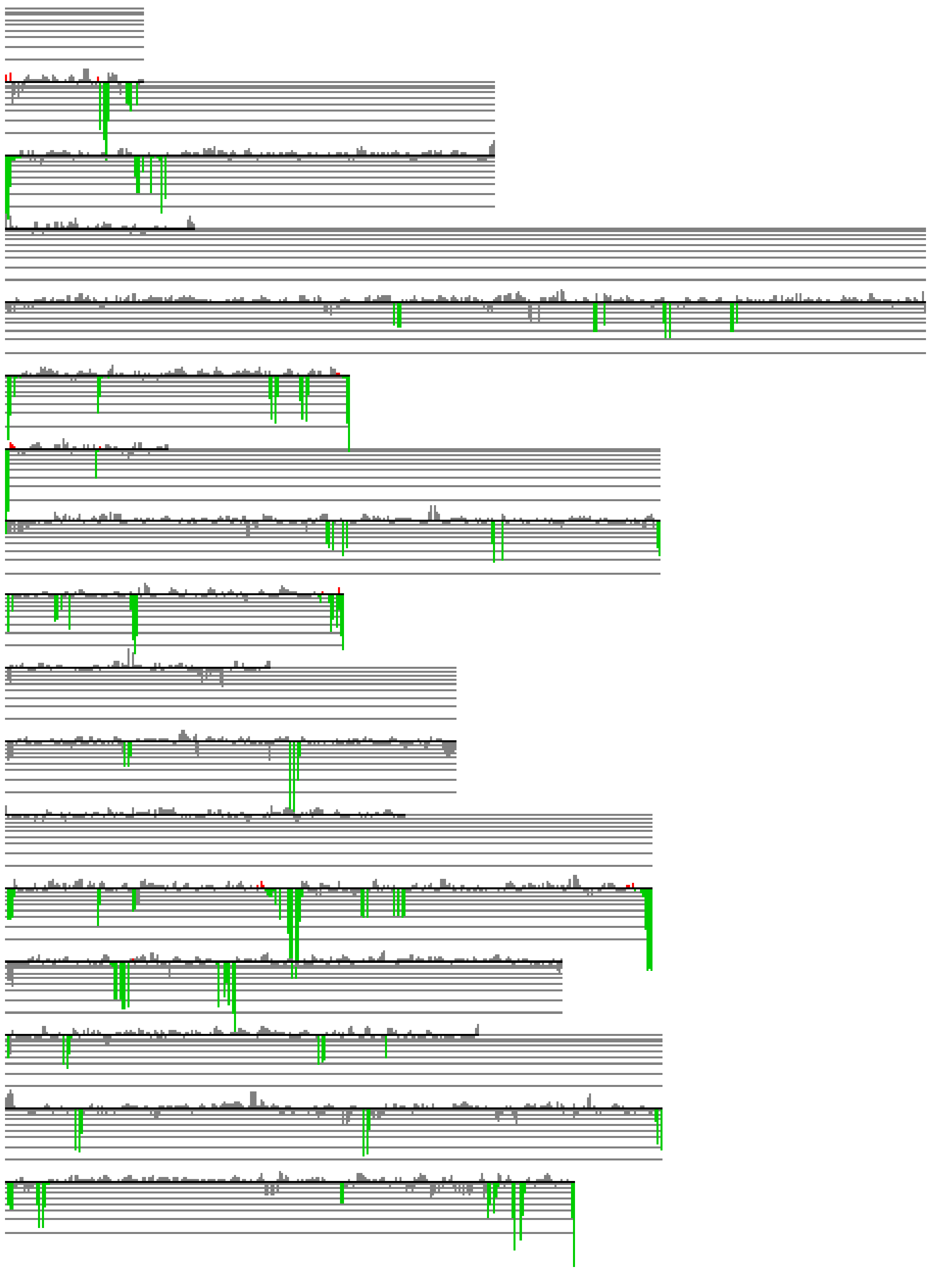

Figure S2O

Karyoscope maps for strain UM218.

For the identification of clusters of ORFs with copy number alterations, the aCGH data was displayed along the chromosomes, using the annotated ORF coordinates of S288C. Vertical bars represent the relative hybridization pattern relatively to the genome of strain S288C. Red bars correspond to amplified ORFs, green bars represent deleted ORFs and grey bars are statistically non-significant alterations. The horizontal lines indicate the hybridization ratios in logarithmic scale. The map was obtained with CGH-Miner, using an averaging moving window of three ORFs.

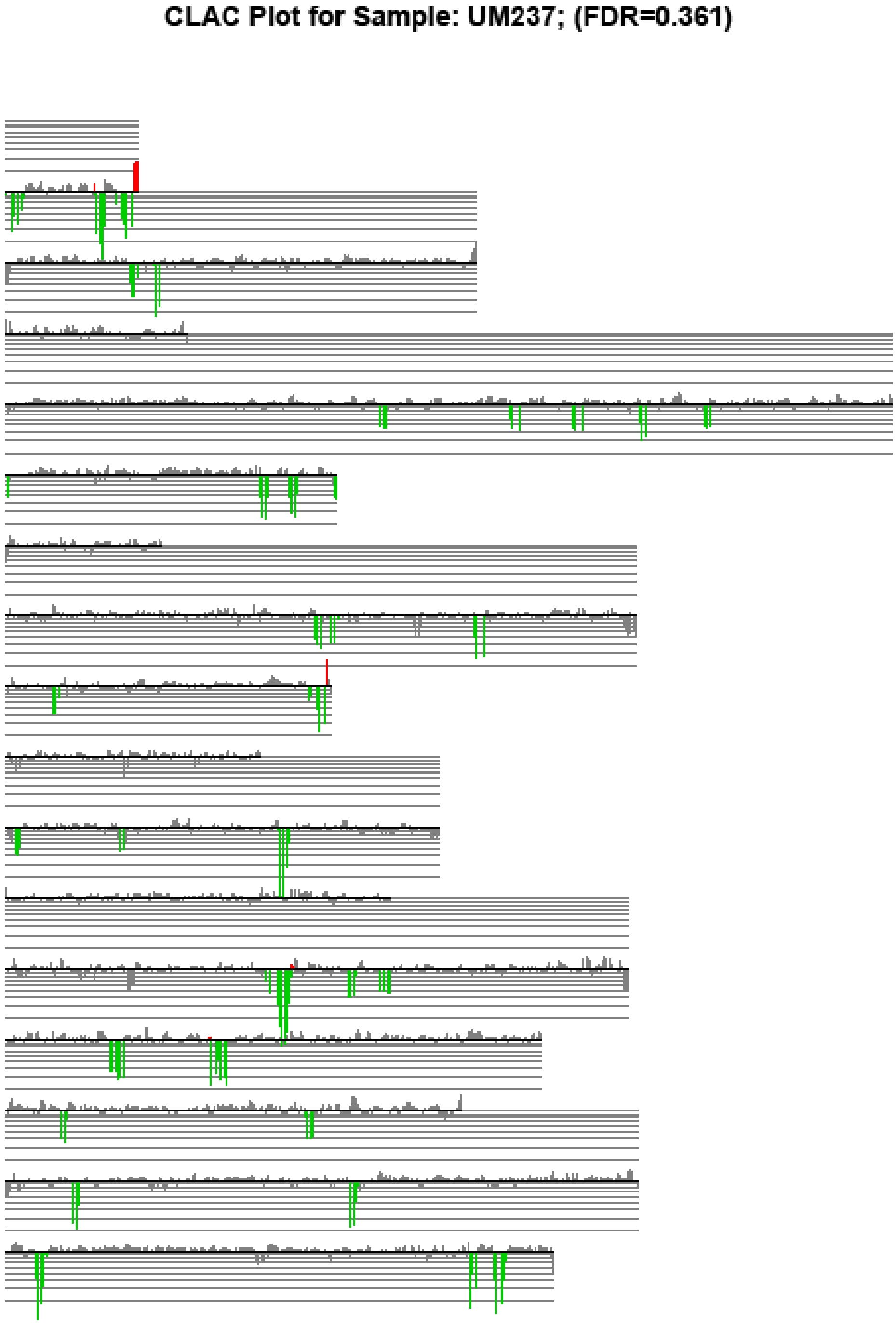

Figure S2P

Karyoscope maps for strain UM237.

For the identification of clusters of ORFs with copy number alterations, the aCGH data was displayed along the chromosomes, using the annotated ORF coordinates of S288C. Vertical bars represent the relative hybridization pattern relatively to the genome of strain S288C. Red bars correspond to amplified ORFs, green bars represent deleted ORFs and grey bars are statistically non-significant alterations. The horizontal lines indicate the hybridization ratios in logarithmic scale. The map was obtained with CGH-Miner, using an averaging moving window of three ORFs.

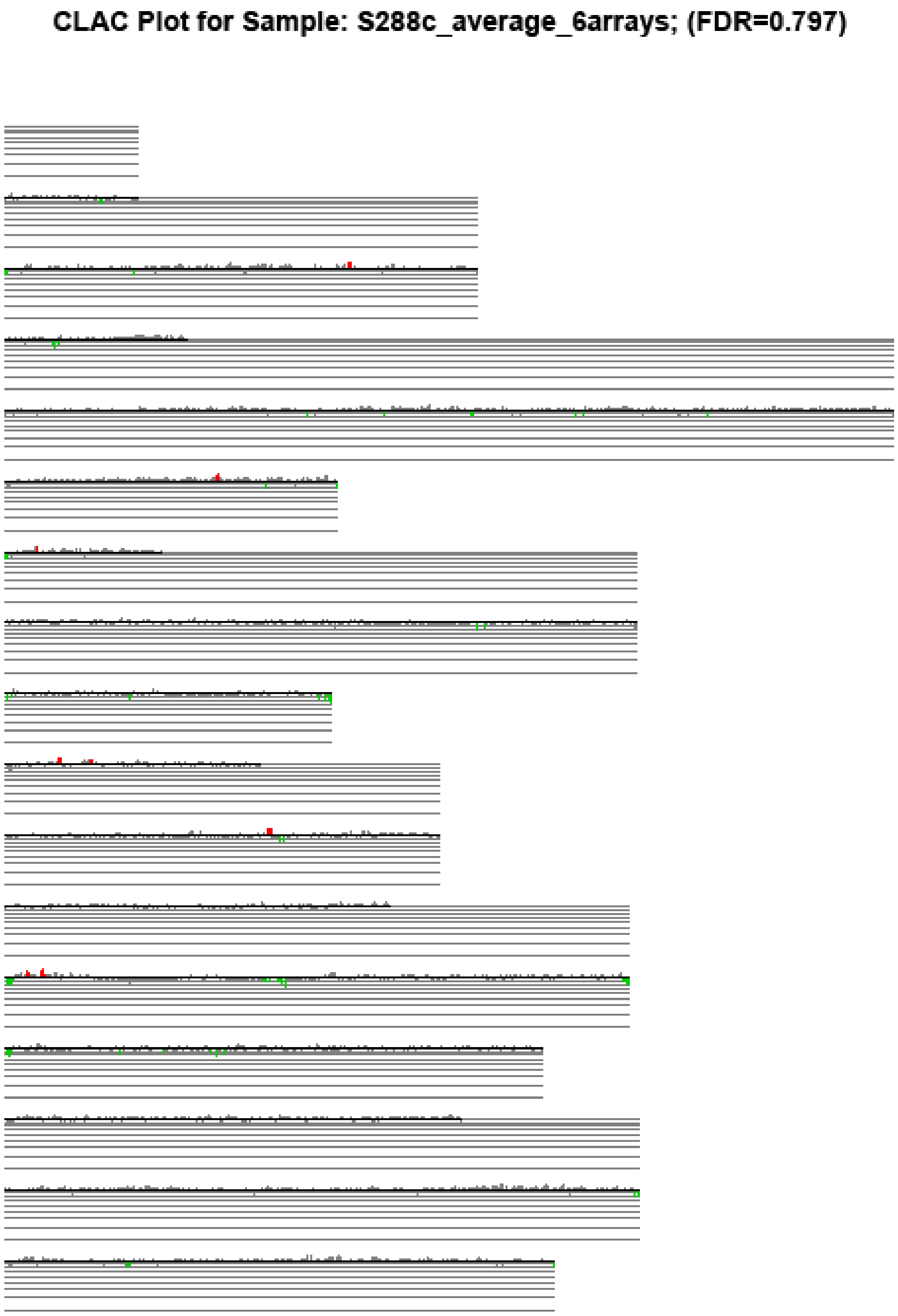

Figure S2Q
Baseline karyoscope
The karyoscope map for strain S288C shows the baseline noise when deriving karyoscope maps for the other analysed strains. It was obtained with the average of six self-self hybridizations performed with the reference strain S288C. The map was obtained with CGH-Miner, using an averaging moving window of three ORFs.
