# Supplementary material for: Comparative genomics of wild type yeast strains unveils important genome diversity
Source: BMC Genomics. 2008 Nov 4;9:524. doi: 10.1186/1471-2164-9-524 (PMC2588607; doi:10.1186/1471-2164-9-524)
Supplement: Additional File 3 — Genome alterations between YJM789 and S288C and this study. Genome alterations found in the consensus plot obtained for the wine strains are compared of the clinical strain YJM789 whose genome is fully sequenced. [file 1471-2164-9-524-S3.pdf]

Chromosome I

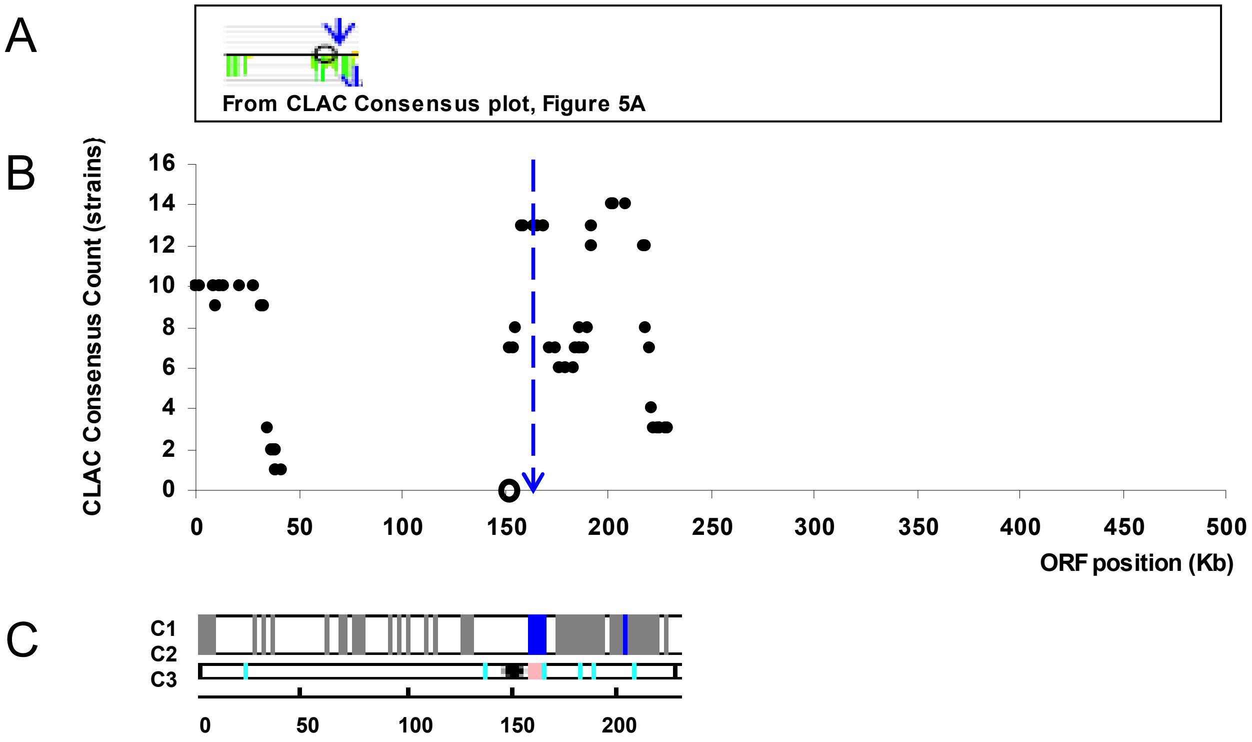

Figure S3A

Comparative analysis of ORF alterations between the wild-type strains used in this study and the YJM789 clinical strain (Wei *et al.*, 2007) in chromosome I.

The genome alterations displayed in the consensus plot of Figure 5A of the manuscript were compared to those observed in the YJM789 clinical strain (Wei *et al.* 2007. *PNAS* 104: 12825-12830). **A)** Map of chromosome I with the coordinates displayed in Figure 5A of the manuscript. The map shows the consensus plot obtained using CGH-Miner. **B)** Graphical representation of the number of strains with copy number alterations for a given ORF. The association between peaks of consensus count and Ty element insertion is highlighted (blue arrow) along the chromosome. The circle in the X-axis indicates the position of the centromere. **C)** The genome sequence alignment between YJM789 and S288C was adjusted to the consensus count plot using the respective chromosome coordinates. **C1**) Display of the ≥100 bp sequences (blue lines) and sequences of <100 bp (gray lines) that are absent in YJM789 but present in S288C. **C2**)The repeat sequences of S288C are represented by colors: cyan rectangles represent long terminal repeats; pink rectangles represent retrotranspons; black rectangles represent telomeres; black circle represents the centromere. **C3)** Coordinates of S288C in Kb pairs. Panel C was adapted from (Wei *et al.*, 2007).

Chromosome II

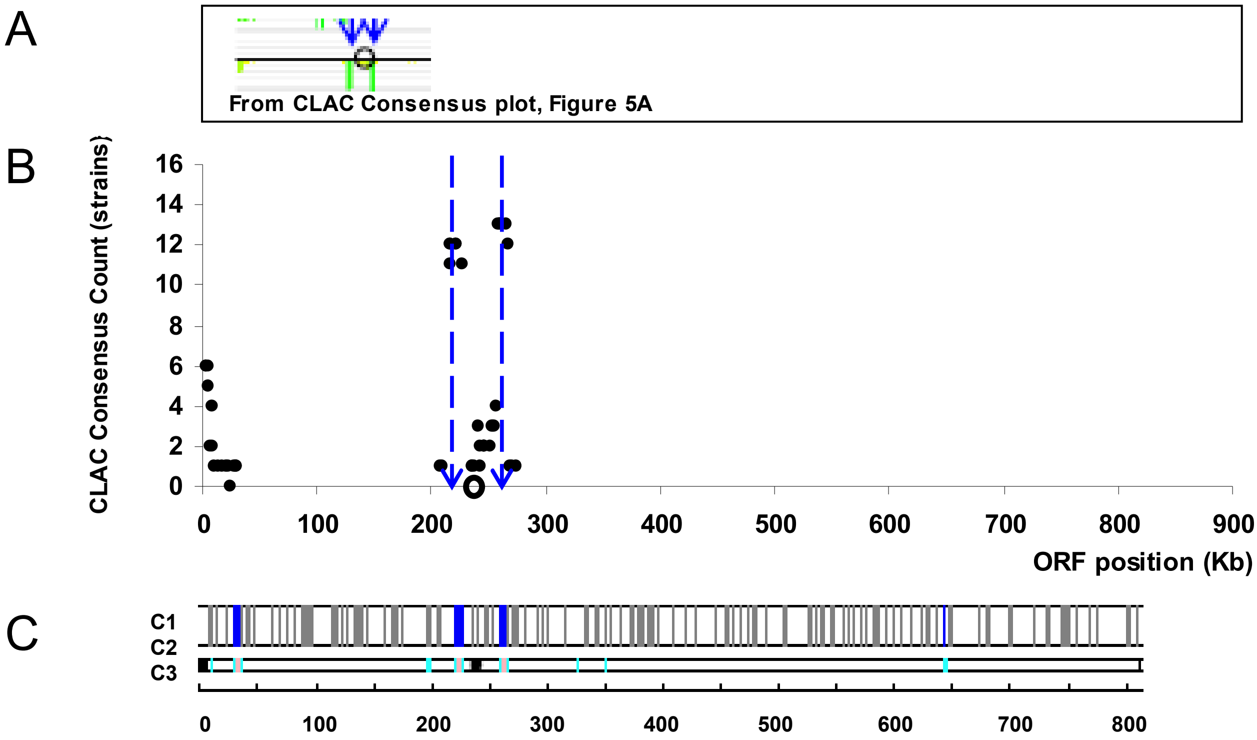

Figure S3B

Comparative analysis of ORF alterations between the wild-type strains used in this study and the YJM789 clinical strain (Wei *et al.*, 2007) in chromosome II. Remaining legend as for Figure S3A.

Chromosome III

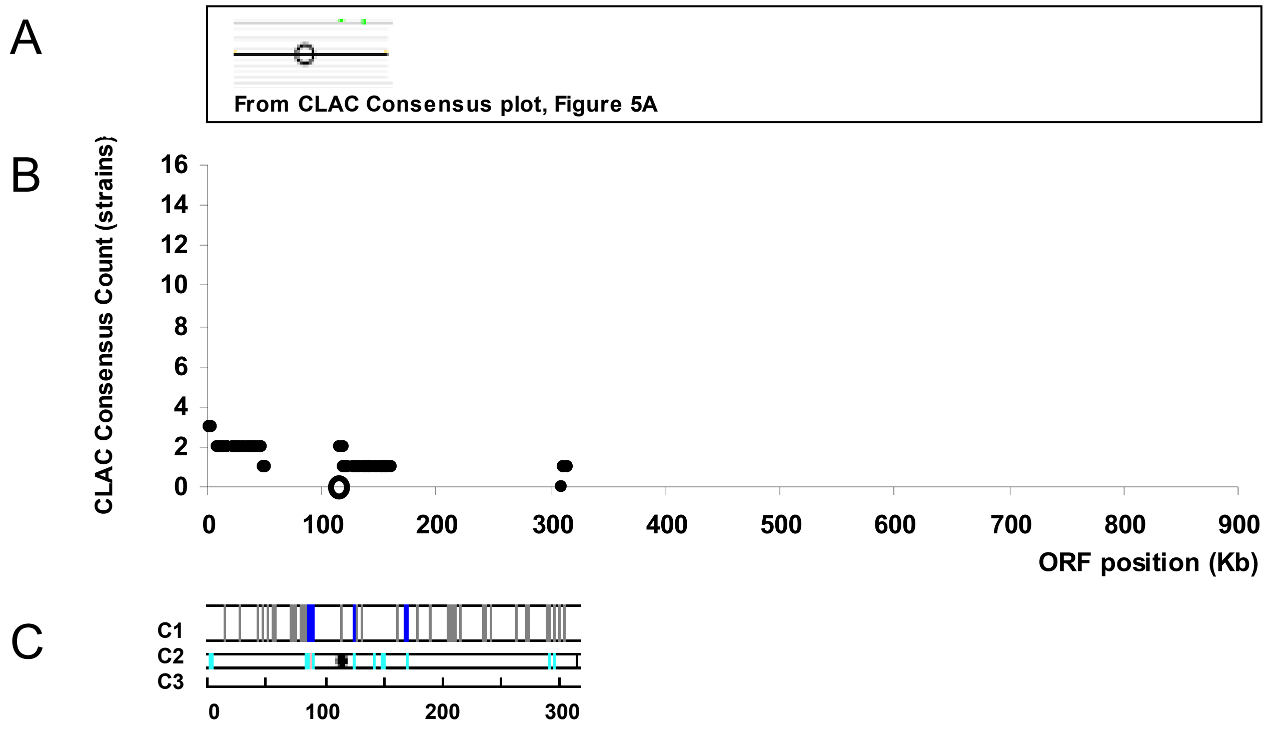

Figure S3C

Comparative analysis of ORF alterations between the wild-type strains used in this study and the YJM789 clinical strain (Wei *et al.*, 2007) in chromosome III. Remaining legend as for Figure S3A.

Chromosome IV

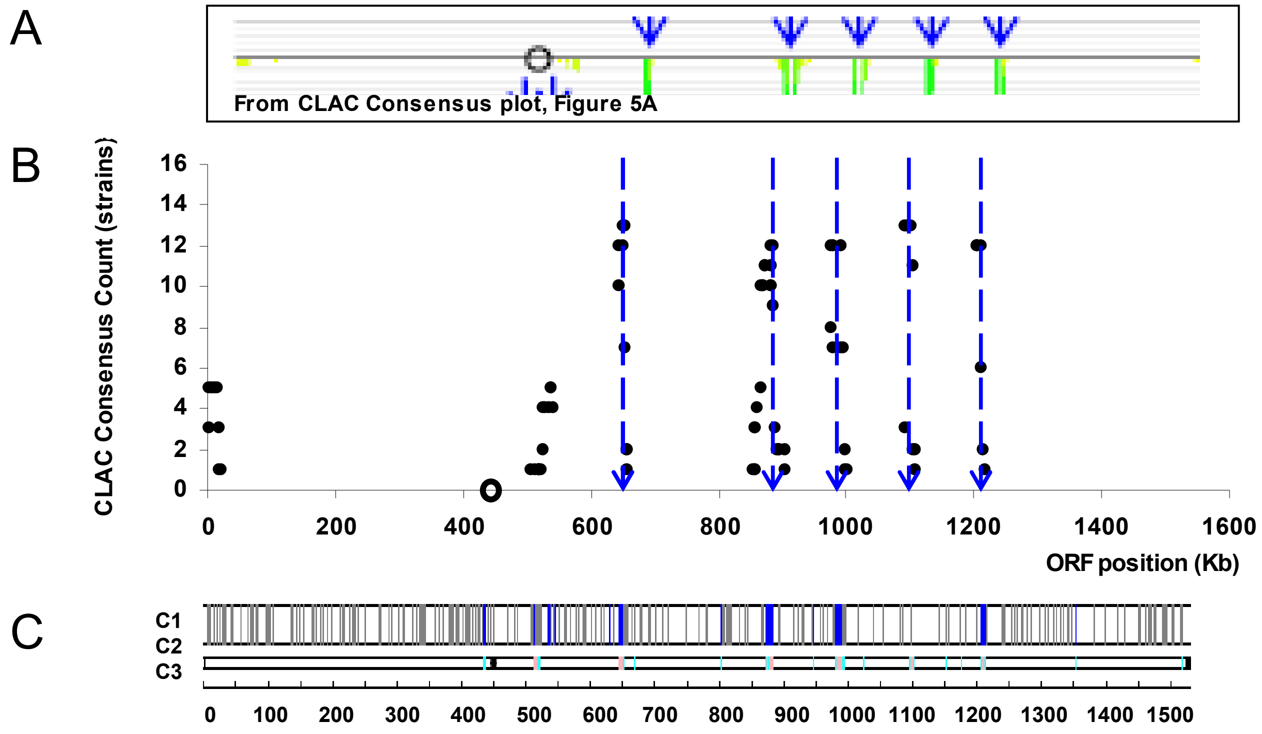

Figure S3D

Comparative analysis of ORF alterations between the wild-type strains used in this study and the YJM789 clinical strain (Wei *et al.*, 2007) in chromosome IV. Remaining legend as for Figure S3A.

Chromosome V

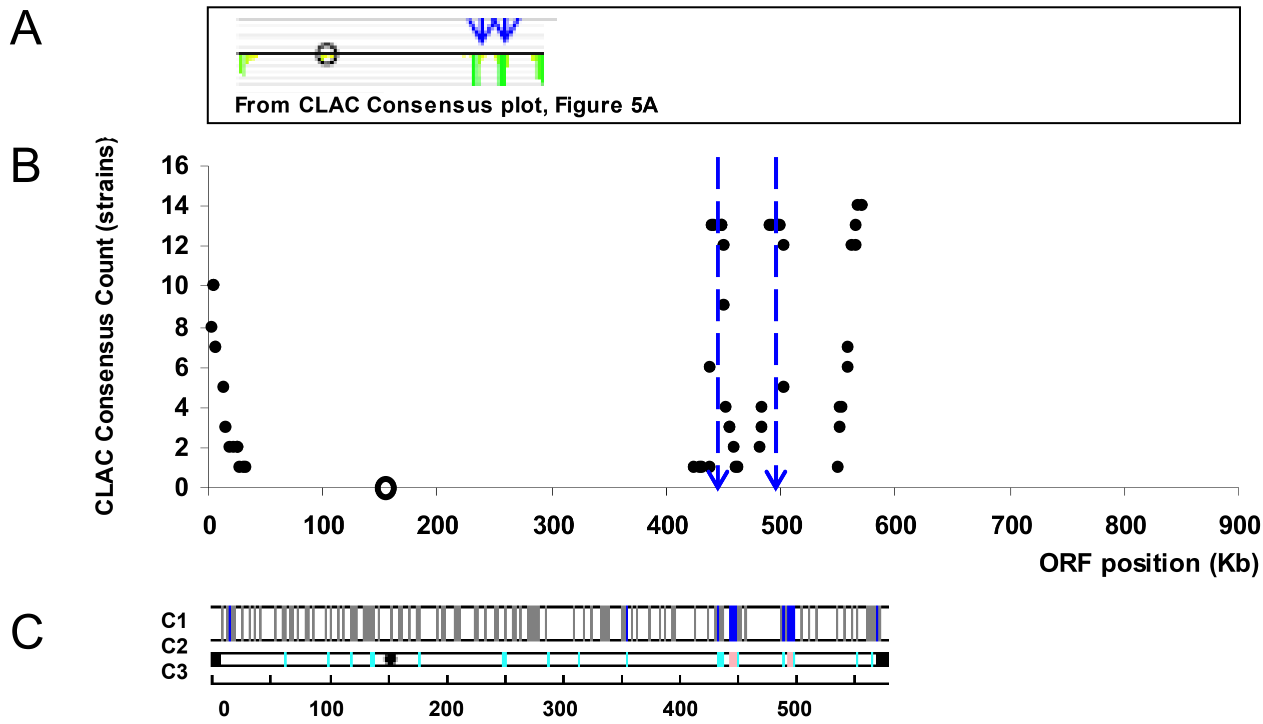

Figure S3E

Comparative analysis of ORF alterations between the wild-type strains used in this study and the YJM789 clinical strain (Wei *et al*., 2007) in chromosome V. Remaining legend as for Figure S3A.

Chromosome VI

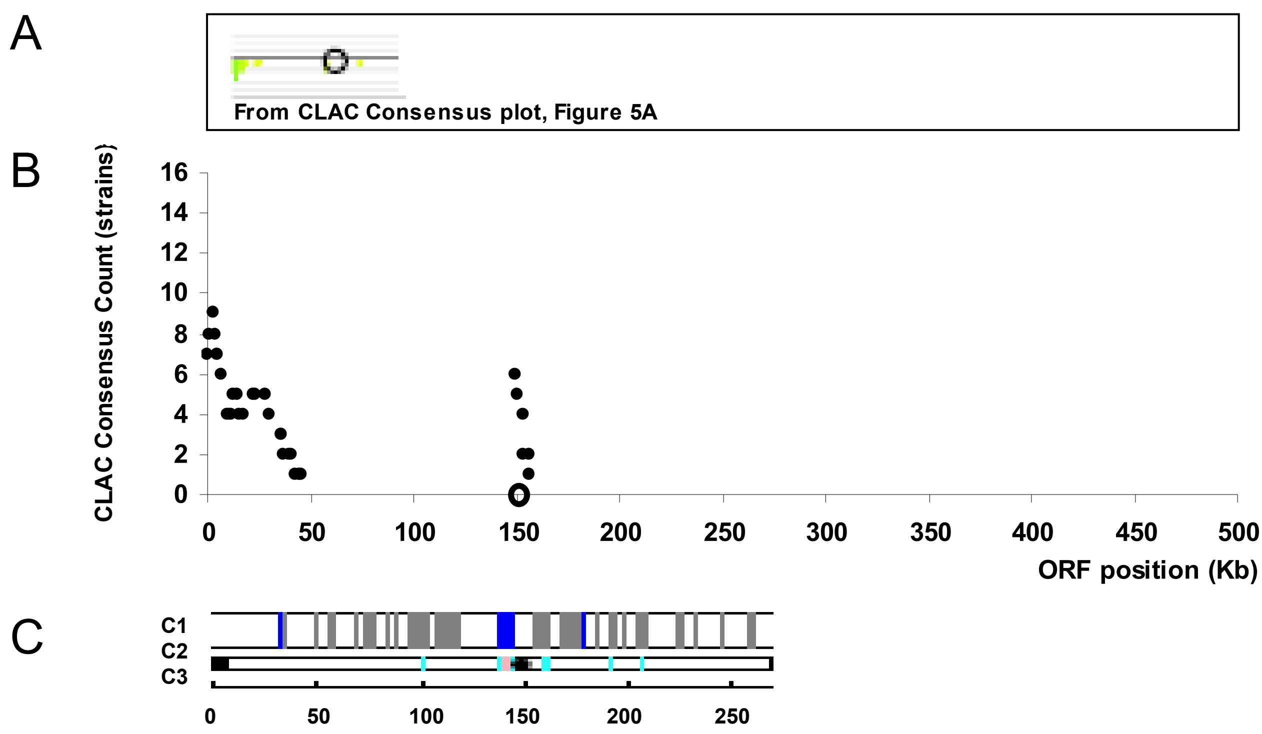

Figure S3F

Comparative analysis of ORF alterations between the wild-type strains used in this study and the YJM789 clinical strain (Wei *et al.*, 2007) in chromosome VI. Remaining legend as for Figure S3A.

Chromosome VII

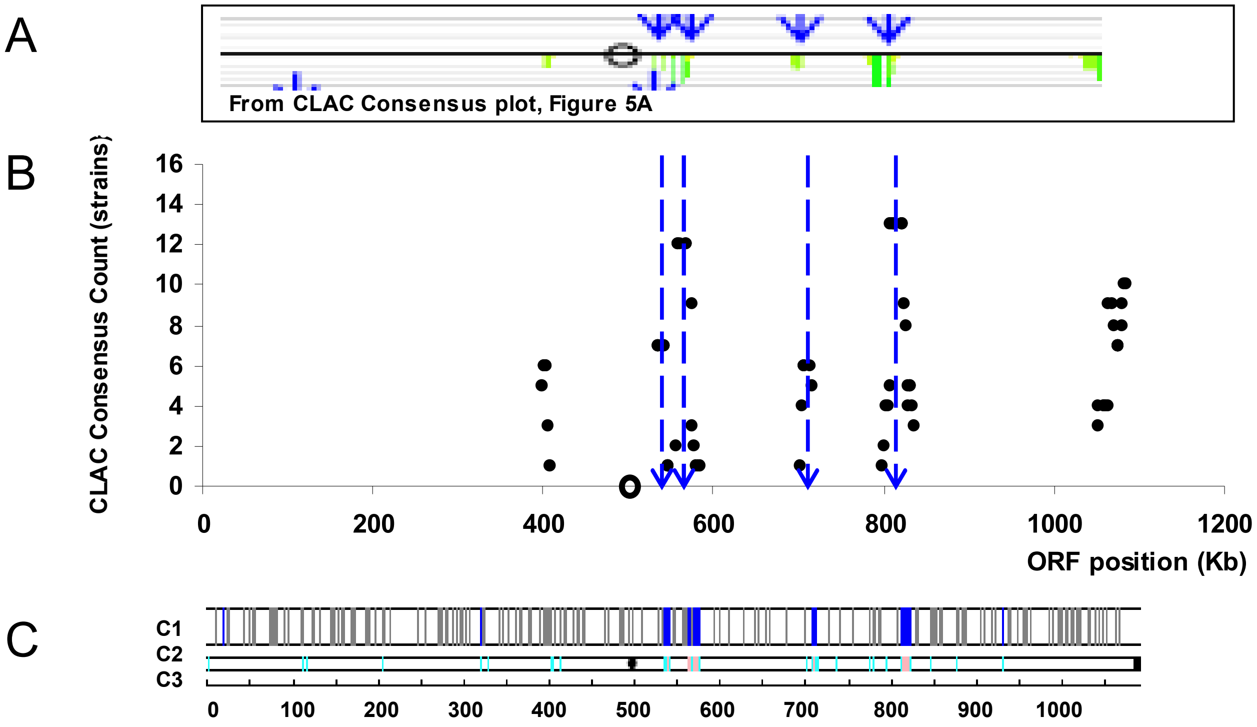

Figure S3G

Comparative analysis of ORF alterations between the wild-type strains used in this study and the YJM789 clinical strain (Wei *et al.*, 2007) in chromosome VII. Remaining legend as for Figure S3A.

Chromosome VIII

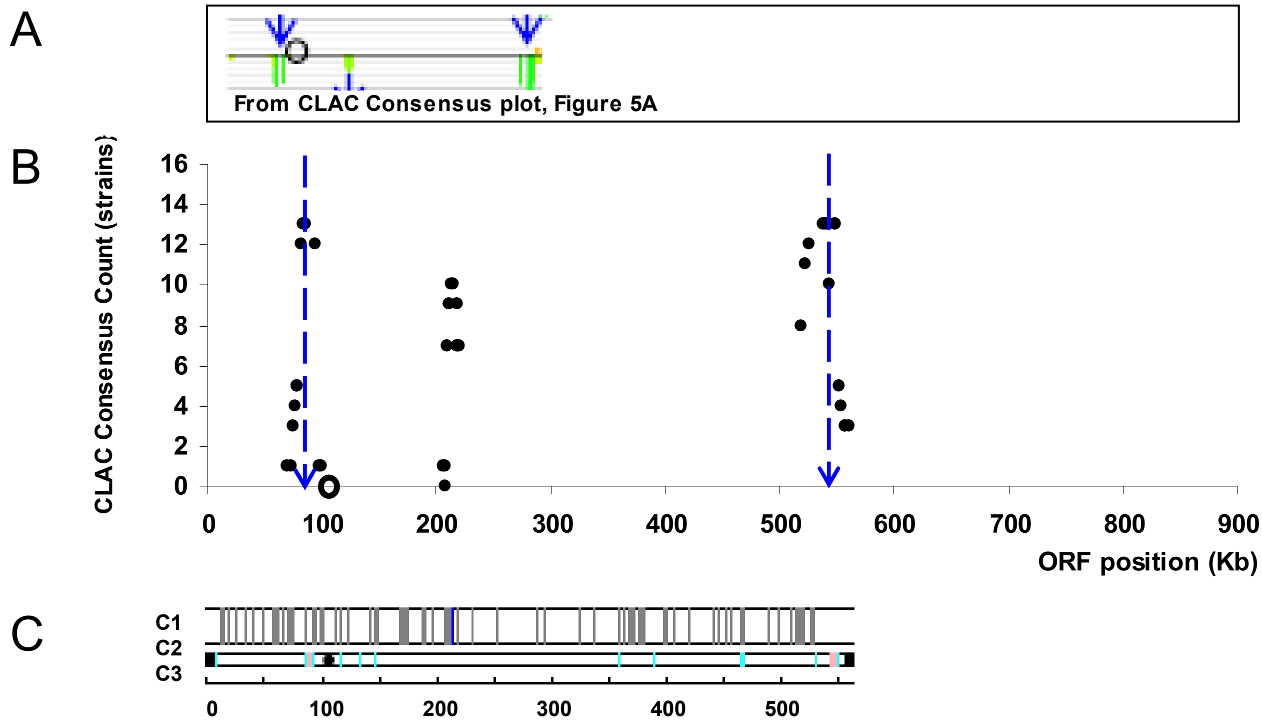

Figure S3H

Comparative analysis of ORF alterations between the wild-type strains used in this study and the YJM789 clinical strain (Wei *et al.*, 2007) in chromosome VIII. Remaining legend as for Figure S3A.

Chromosome IX

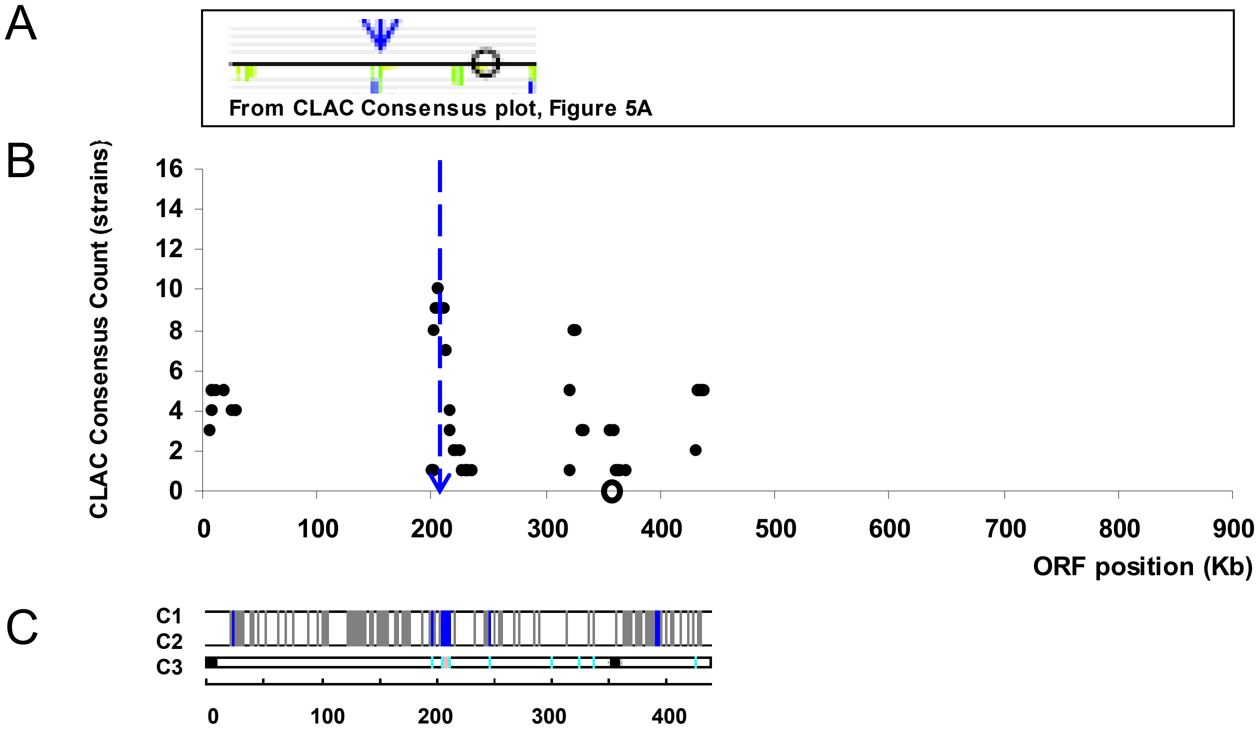

Figure S3I
Comparative analysis of ORF alterations between the wild-type strains used in this study and the YJM789 clinical strain (Wei *et al.*, 2007) in chromosome IX. Remaining legend as for Figure S3A.

Chromosome X

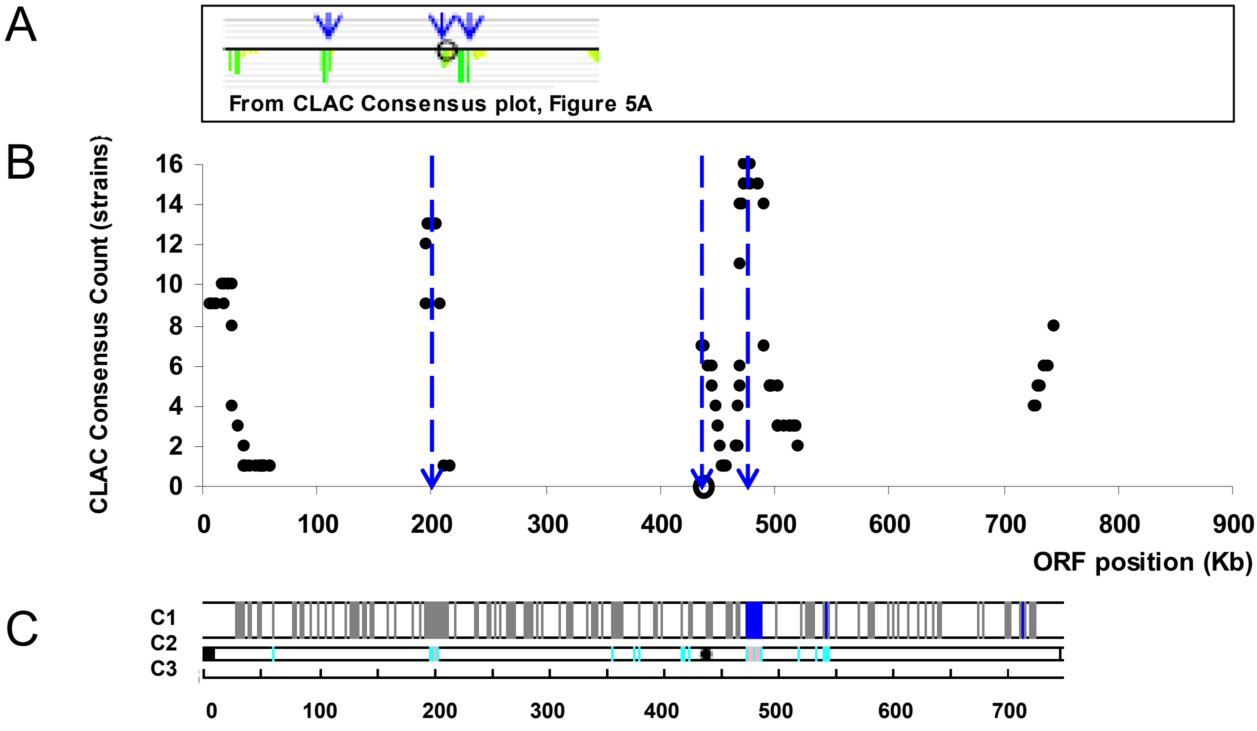

Figure S3J

Comparative analysis of ORF alterations between the wild-type strains used in this study and the YJM789 clinical strain (Wei *et al.*, 2007) in chromosome X. Remaining legend as for Figure S3A.

Chromosome XI

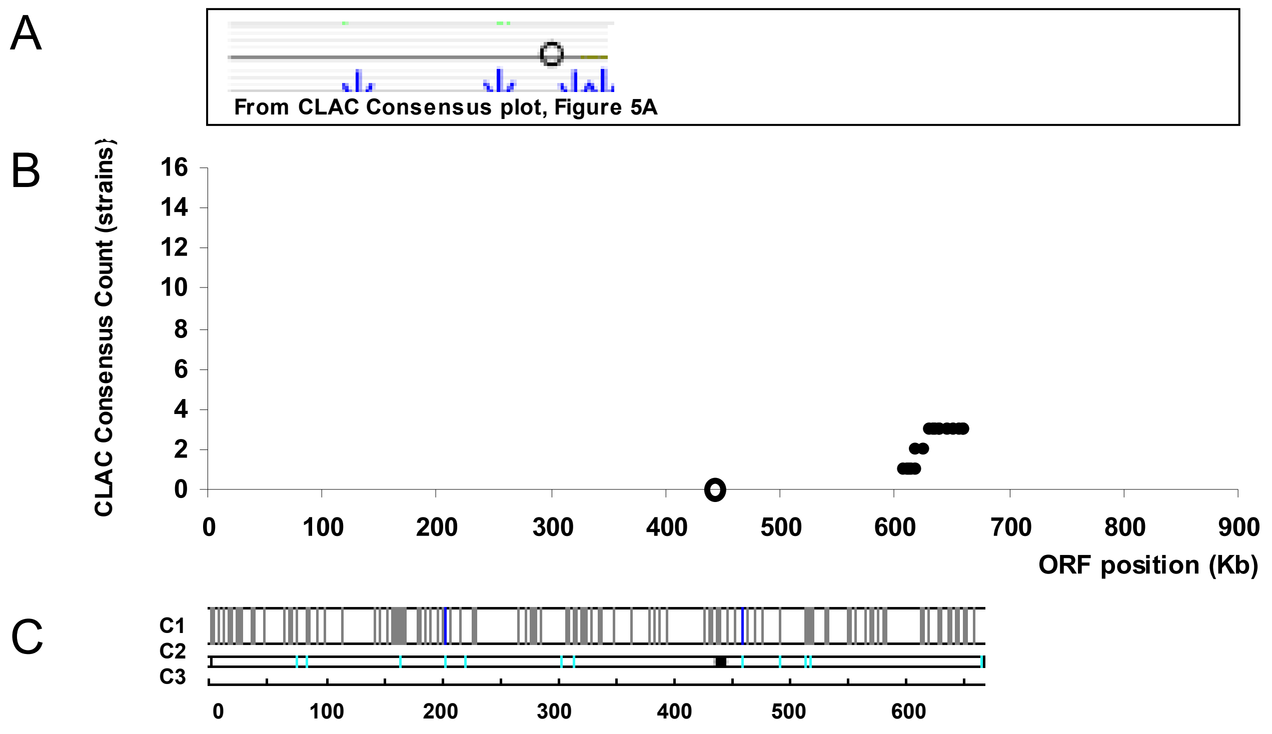

Figure S3K

Comparative analysis of ORF alterations between the wild-type strains used in this study and the YJM789 clinical strain (Wei *et al.*, 2007) in chromosome XI. Remaining legend as for Figure S3A.

Chromosome XII

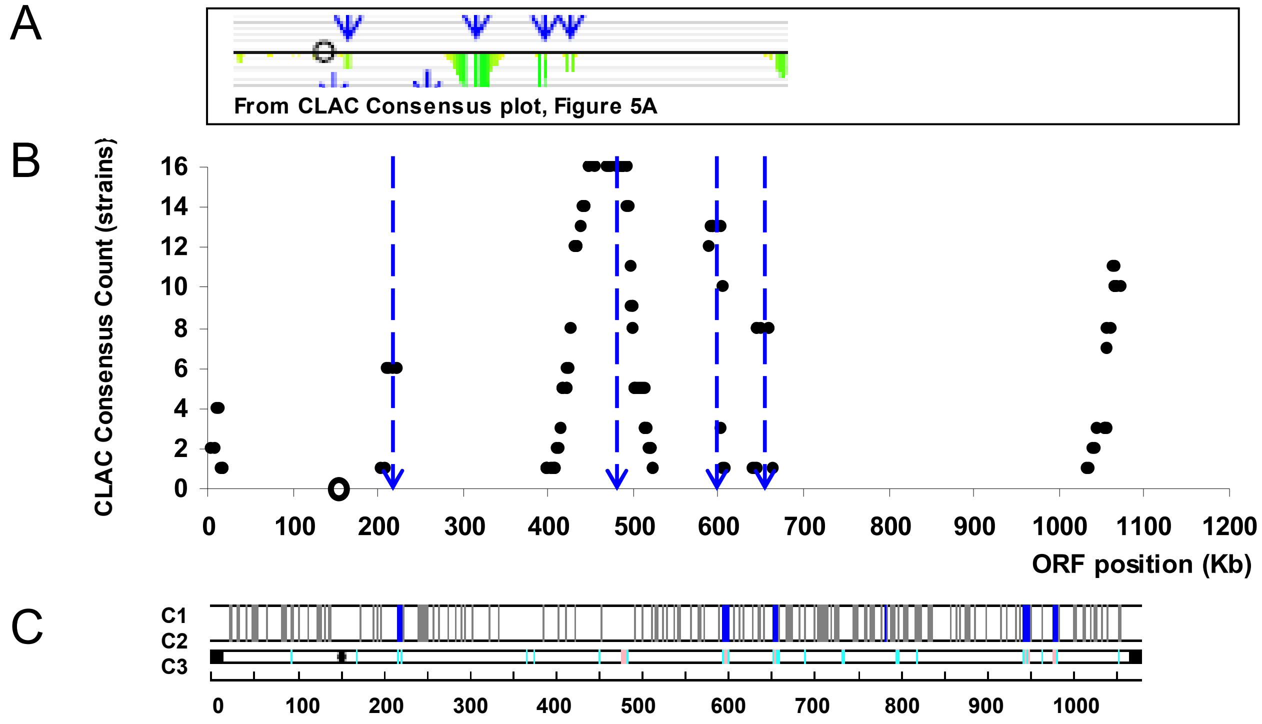

Figure S3L
Comparative analysis of ORF alterations between the wild-type strains used in this study and the YJM789 clinical strain (Wei *et al.*, 2007) in chromosome XII. Remaining legend as for Figure S3A.

Chromosome XIII

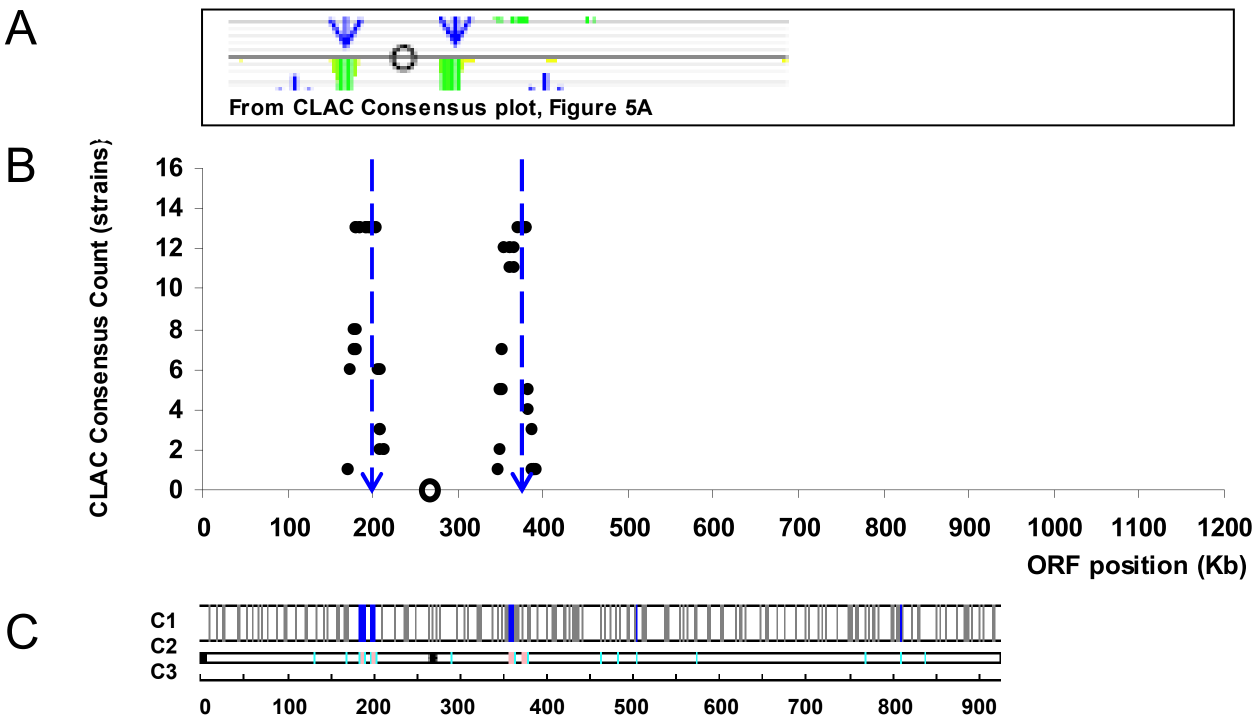

Figure S3M

Comparative analysis of ORF alterations between the wild-type strains used in this study and the YJM789 clinical strain (Wei *et al.*, 2007) in chromosome XIII. Remaining legend as for Figure S3A.

Chromosome XIV

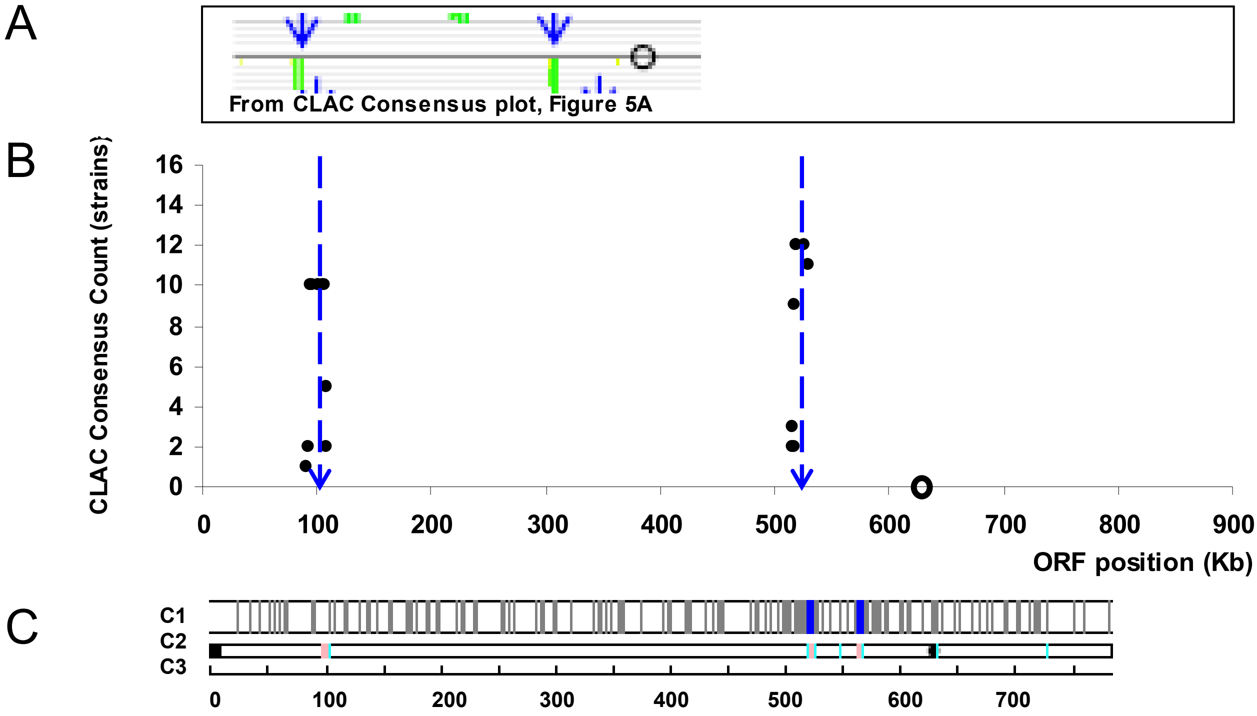

Figure S3N

Comparative analysis of ORF alterations between the wild-type strains used in this study and the YJM789 clinical strain (Wei *et al.*, 2007) in chromosome XIV. Remaining legend as for Figure S3A.

Chromosome XV

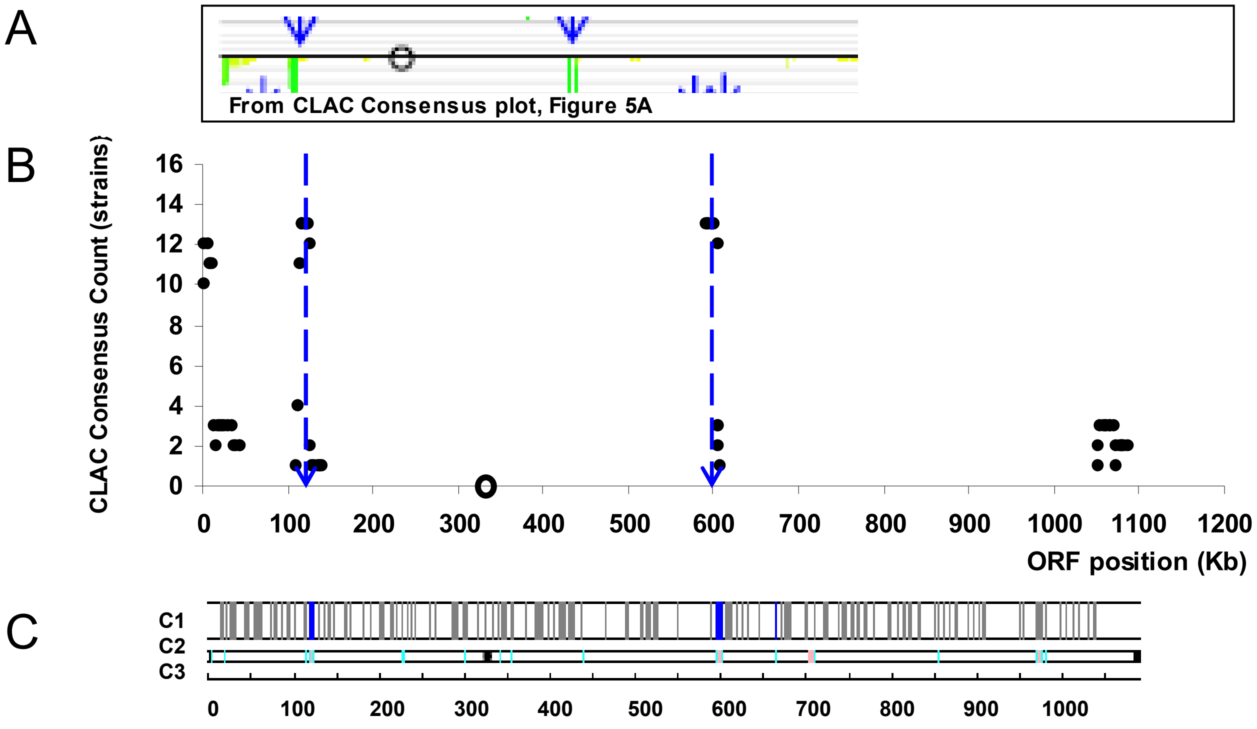

Figure S3O

Comparative analysis of ORF alterations between the wild-type strains used in this study and the YJM789 clinical strain (Wei *et al.*, 2007) in chromosome XV. Remaining legend as for Figure S3A.

Chromosome XVI

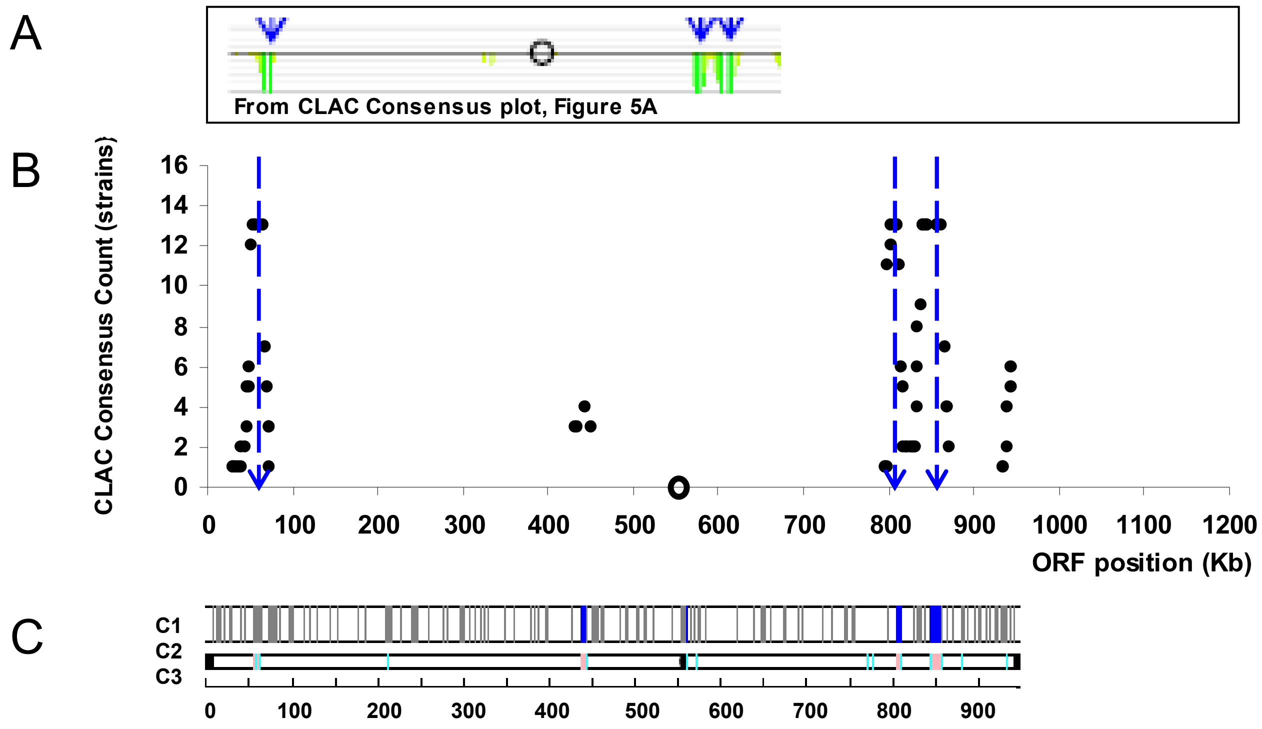

Figure S3P

Comparative analysis of ORF alterations between the wild-type strains used in this study and the YJM789 clinical strain (Wei *et al.*, 2007) in chromosome XVI. Remaining legend as for Figure S3A.
